# Effect of Welding Heat Input on Microstructure and Properties of CGHAZ in Deep-Sea Oil and Gas Transportation Pipeline Steel

**DOI:** 10.3390/ma19163382

**Published:** 2026-08-08

**Authors:** Lili Ran, Shilin Liu, Ba Li, Yanan Li, Rui Hong, Bing Wang, Qingyou Liu, Shujun Jia

**Affiliations:** 1 Department of Structural Steels, Central Iron and Steel Research Institute Co., Ltd., Beijing 100081, China; 2 Construction Project Management Branch of China Oil and Gas Pipeline Network Group Co., Ltd., Langfang 065001, China; 3 CNPC Baoji Petroleum Pipe Industry Co., Ltd., Baoji 721008, China; 4 Institute of Research of Iron and Steel, Jiangsu Province/Sha-Steel Co., Ltd., Zhangjiagang 215625, China

**Keywords:** deep-sea oil and gas transportation pipeline steel, welding heat input, CGHAZ, M/A constituent, low-temperature toughness

## Abstract

Gleeble-3800 thermal simulation testing machine was adopted to investigate the evolution laws of microstructure and properties in the coarse-grained heat-affected zone (CGHAZ) of pipeline steels with different Cr mass fractions (0.2, 0.5, 0.8 wt.%) under welding heat inputs ranging from 8 kJ/cm to 20 kJ/cm. Combined with optical microscopy, scanning electron microscopy and electron backscatter diffraction, the coupled influencing mechanism of heat input and Cr content on the properties of CGHAZ was systematically analyzed. The results show that as the Cr content increases, the range of valid welding heat input for maintaining satisfactory CGHAZ impact toughness gradually narrows with increasing Cr mass fraction of the steel. Specifically, the 0.2Cr experimental steel maintains high toughness under a thermal input ranging from 8 to 20 kJ/cm, the 0.5Cr experimental steel exhibits relatively high toughness in the range 8 to 13 kJ/cm, while the 0.8Cr experimental steel shows high toughness only at 15 kJ/cm. The coupled effect of weld heat input and Cr content on CGHAZ properties originates from a combination of microstructural composition types, phase fractions, substructures, and grain sizes. Increasing the heat input and Cr content leads to a reduction in the bainite ferrite with superior toughness and an increase in the large-sized granular bainite with inferior toughness. Meanwhile, the effective grain size of the overall microstructure first decreases and then rises. Grain coarsening and an increased fraction of Martensitic/Austenitic (M/A) constituent are the key factors responsible for the deterioration of CGHAZ toughness in deep-sea oil and gas transportation pipeline steel.

## 1. Introduction

With the continuous growth of global energy demand, the exploitation of deep-sea oil and gas resources has gradually become a key area of the energy strategy [1]. Nevertheless, the service environment for deep-sea oil and gas transportation is extremely harsh. The medium inside the pipeline generally contains impurities such as CO_2_ and free water, which react with each other to generate carbonic acid and trigger severe CO_2_ corrosion on the inner wall of the pipeline steel. Particularly when other impurities, including H_2_S, exist in the medium, the corrosion intensity rises drastically, even resulting in corrosion perforation or cracking failure, which seriously endangers the safe operation and service life of pipelines [2,3,4]. Accordingly, it is urgent to develop special pipeline steels for deep-sea oil and gas transportation with matched high strength-toughness and outstanding corrosion resistance.

Alloying with Cr has been verified as an effective and economical method to enhance the CO2 corrosion resistance of pipeline steel [5,6,7]. Previous studies [8,9,10,11,12] reveal that appropriate Cr addition facilitates the generation of dense and stable Cr-rich corrosion product films (e.g., amorphous Cr(OH)_3_ or Cr_2_O_3_) on the inner pipe surface, remarkably reducing the corrosion rate and delaying the corrosion-induced failure of materials. Nevertheless, excessive Cr content markedly impairs the toughness and weldability of pipeline steel, manifested as degraded low-temperature toughness of the welding heat-affected zone, elevated cold cracking susceptibility, and deteriorated plasticity and toughness of weld metal, while raising the difficulty of welding process control simultaneously [13,14,15,16]. Hence, optimizing Cr content and corresponding welding processes to improve welding quality on the premise of guaranteed corrosion resistance is one of the critical research topics for deep-sea oil and gas transportation pipeline steels.

Scholars at home and abroad have carried out relevant investigations aiming at the above issues [17,18]. For corrosion performance, most experimental steels adopted in existing research contain Cr no more than 5 wt.%, and the stabilizing effect of Cr on CO_2_ corrosion products has been validated. However, systematic research concerning the weldability of pipeline steels with varying Cr contents remains insufficient. Most published literature only focuses on welding process matching or corrosion behavior of welded joints under a single Cr content, lacking comparative analysis on the correlation rules among microstructure evolution, hardness distribution and impact toughness of the heat-affected zone with different Cr contents [19]. Moreover, as a strong carbide-forming element, Cr alters the continuous cooling transformation behavior of the coarse-grained heat-affected zone (CGHAZ) in pipeline steel under non-equilibrium welding thermal cycles [20]. It has been reported [21] that higher Cr content continuously improves corrosion resistance but elevates the hardness and deteriorates the low-temperature impact toughness of CGHAZ, which can be attributed to the prominent modification of prior austenite grain size and phase transformation products in CGHAZ induced by Cr.

Typically, pipeline materials with 1–5 wt.% Cr content are used in limited quantities in branch lines of onshore pipeline networks, whereas the Cr content in mainline pipeline steels is generally ≤0.3 wt.%. Our team has successively investigated the effects of 0.1–0.5 wt.% Cr on the microstructure and properties of the heat-affected zone (HAZ) in X80 high-grade pipeline steel and supercritical CO_2_ transmission pipeline steel [22]. However, research on the coupled effects of higher Cr content (0.5–1 wt.%) pipeline steels and welding processes for deep-sea environments has not yet been reported.

In this work, the welding performance of pipeline steels with Cr contents ranging from 0.21 wt.% to 0.81 wt.% under different welding heat inputs is systematically investigated. Welding thermal simulation tests, mechanical property measurements and multiple material characterization techniques are employed to analyze the coupled influence of heat input and Cr content on microstructure evolution, hardness distribution and low-temperature toughness of the heat-affected zone. Combined with microstructural characterization, the action mechanism of Cr is clarified. The present study provides theoretical foundations and engineering guidance for composition optimization design and welding process formulation of corrosion-resistant pipeline steels for deep-sea oil and gas transportation.

## 2. Experimental Materials and Methods

### 2.1. Experimental Materials

The experimental materials adopted in this study are three kinds of laboratory-rolled pipeline steels with different Cr contents, and their chemical compositions are listed in Table 1.

### 2.2. Experimental Methods

**Figure 1:**
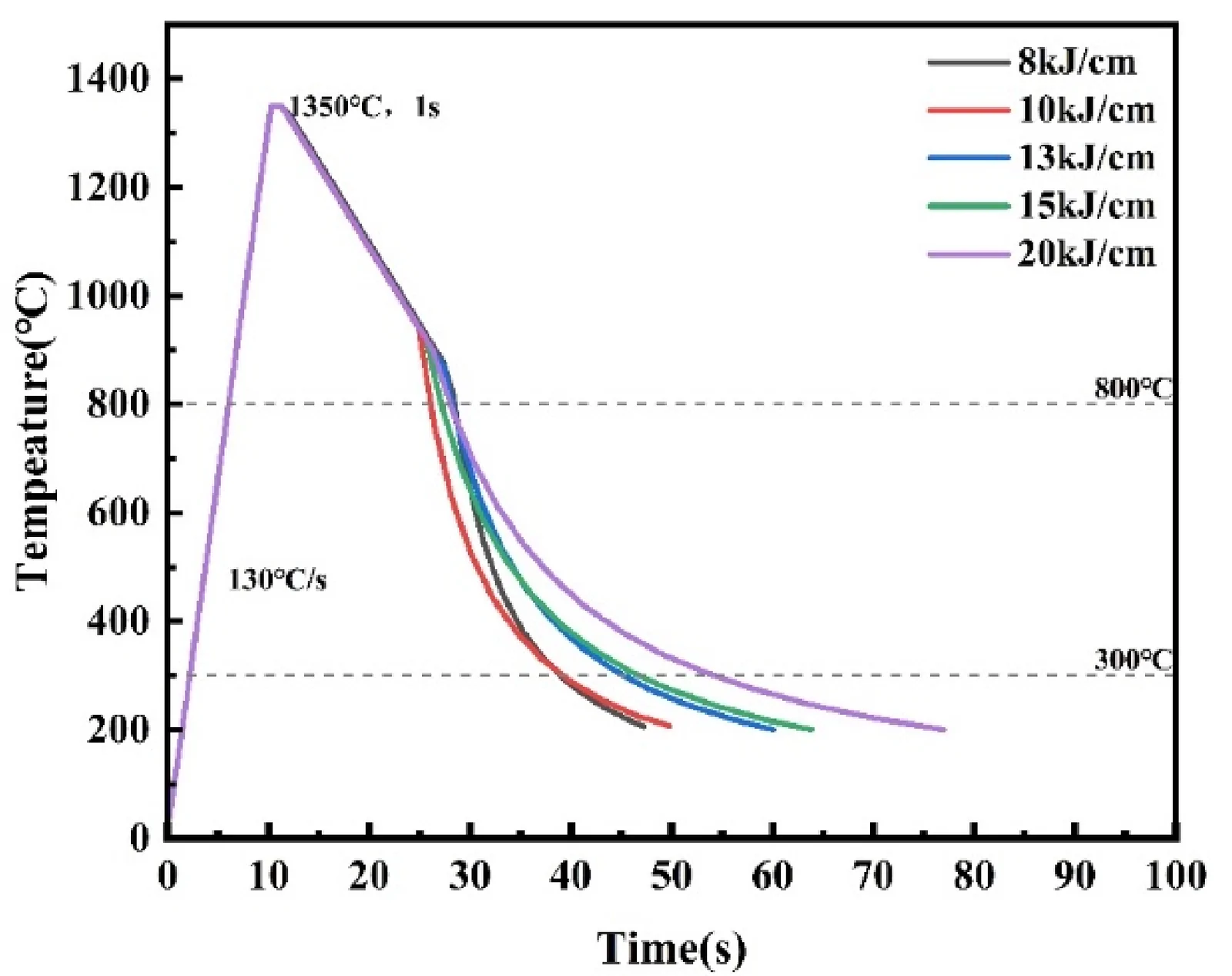
Thermal cycling curve of CGHAZ under different heat input.

In this study, a Gleeble-3800 thermomechanical simulation tester (Dynamic Systems Inc., Poestenkill, NY, USA) was employed to conduct welding thermal simulation tests on the experimental steel. Specimens with dimensions of 10.5 mm × 10.5 mm × 60 mm were machined along the rolling direction for thermal simulation experiments. Before testing, the equipment was calibrated for welding thermal simulation. All specimens were heated to 1300 °C at a heating rate of 130 °C/s and held for 1 s, followed by continuous cooling to 800 °C. Subsequently, the specimens were cooled down to 300 °C under different welding heat inputs and finally cooled naturally to room temperature. The thermal simulation parameters of the coarse-grained heat-affected zone (CGHAZ) under various welding heat inputs are listed in Table 2, and the corresponding thermal cycle curves are presented in Figure 1. All thermal cycle parameters corresponding to the heat inputs ranging from 8 to 20 kJ/cm were calculated based on the classical Rosenthal thick-plate heat conduction model. This model is widely applicable to the welding thermal simulation of medium and thick pipeline steels, ensuring high accuracy in the conversion of thermal cycle parameters.

Most existing published relevant studies take T_8/5_ (the cooling time from 800 °C to 500 °C) as the key control parameter for thermal simulation, whereas the present work selects T_8/3_ (the cooling time from 800 °C to 300 °C) as the primary governing variable. This parameter selection is comprehensively determined based on steel phase transformation theory and actual on-site welding service conditions. The T_8/3_ index covers the complete cooling window from 800 °C to 300 °C, which fully encompasses the entire temperature range for bainite transformation. It can faithfully reproduce the whole evolution process of the coarse-grained heat-affected zone (CGHAZ) during welding, including the decomposition of high-temperature austenite, continuous precipitation of bainite, and the termination of bainitic transformation. Therefore, the thermal simulation scheme controlled by T_8/3_ achieves higher fidelity to the actual thermal history of field welding, and the corresponding test results possess superior accuracy for mechanism analysis and engineering reference compared with the conventional simulation strategy merely utilizing T_8/5_.

Three Charpy impact specimens with dimensions of 55 mm × 10 mm × 10 mm were fabricated for each group, and the instrumented impact tests were conducted in accordance with the standard GB/T 19748-2019 [23]. Two cylindrical tensile specimens with a diameter of 10 mm were machined along the rolling direction for each group, and room-temperature tensile tests were performed on an Instron-5958 universal electro-mechanical testing machine following the standard GB/T 228-2021 [24].

Metallographic specimens sized 30 mm × 20 mm × 20 mm were cut from the welded joints. An FEI Quanta 650 FEG field-emission scanning electron microscope (FEI, Hillsboro, OR, USA) was adopted to characterize the microstructure morphology and impact fracture surfaces of the steels. Ten discrete points at different positions in the specimen core were selected for Vickers microhardness measurement using an FM300 microhardness tester (Future-Tech Corp., Kawasaki, Japan). The central region of the welded metallographic samples was electrolytically polished in a 10 vol.% perchloric acid alcohol solution. The impact fracture surfaces were nickel-plated, followed by vibratory polishing. EBSD characterization was implemented on the field-emission SEM equipped with an Oxford F-plus EBSD detector (Oxford Instruments, Abingdon, UK).

## 3. Results

### 3.1. Microstructure and Mechanical Properties of Experimental Steel-Based Materials with Different Cr Contents

The mechanical properties of the three experimental steels are summarized in Table 3, and Rt_0.5_ represents the 0.5% offset yield strength, Rm denotes the ultimate tensile strength, Rt_0.5_/R_m_ is the yield-to-tensile strength ratio, A/% refers to the elongation after fracture, and HV_0.5_ indicates the Vickers hardness tested under a load of 0.5 kgf. With increasing Cr content, the overall strength and hardness of the tested steels rise, while the impact toughness declines, and all steels exhibit favorable strength-toughness matching.

**Figure 2:**
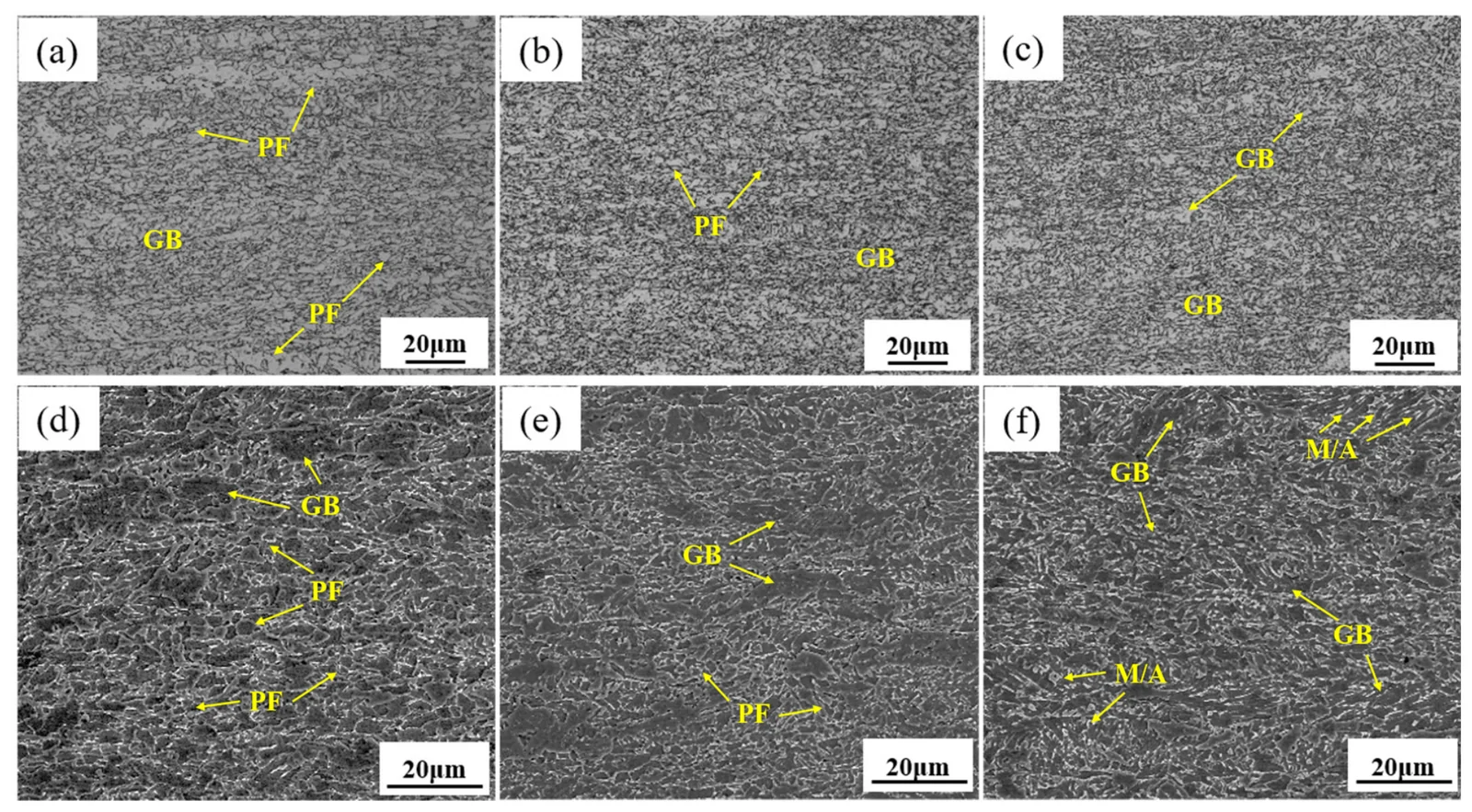
Microstructure of the longitudinal center of experimental steel: (**a**) 0.2Cr steel optical microscopy; (**b**) 0.5Cr steel optical microscopy; (**c**) 0.8Cr steel optical microscopy; (**d**) 0.2Cr steel SEM imaging; (**e**) 0.5Cr steel SEM imaging; (**f**) 0.8Cr steel SEM imaging.

Figure 2 presents optical metallographic and SEM micrographs of the three experimental steels, whose microstructures are mainly composed of polygonal ferrite (PF) and granular bainite (GB). It can be observed that the fraction of GB rises with increasing Cr content, accompanied by a higher volume fraction of Martensitic/Austenitic (M/A) constituents embedded within GB. This phenomenon enhances the strength of the granular bainite phase in the 0.8Cr steel, leading to elevated ultimate tensile strength and hardness of the overall microstructure. Nevertheless, the hardness difference between PF and GB becomes larger, which reduces the yield-to-tensile strength ratio.

### 3.2. Effect of Welding Heat Input on Mechanical Properties of CGHAZ

**Figure 3:**
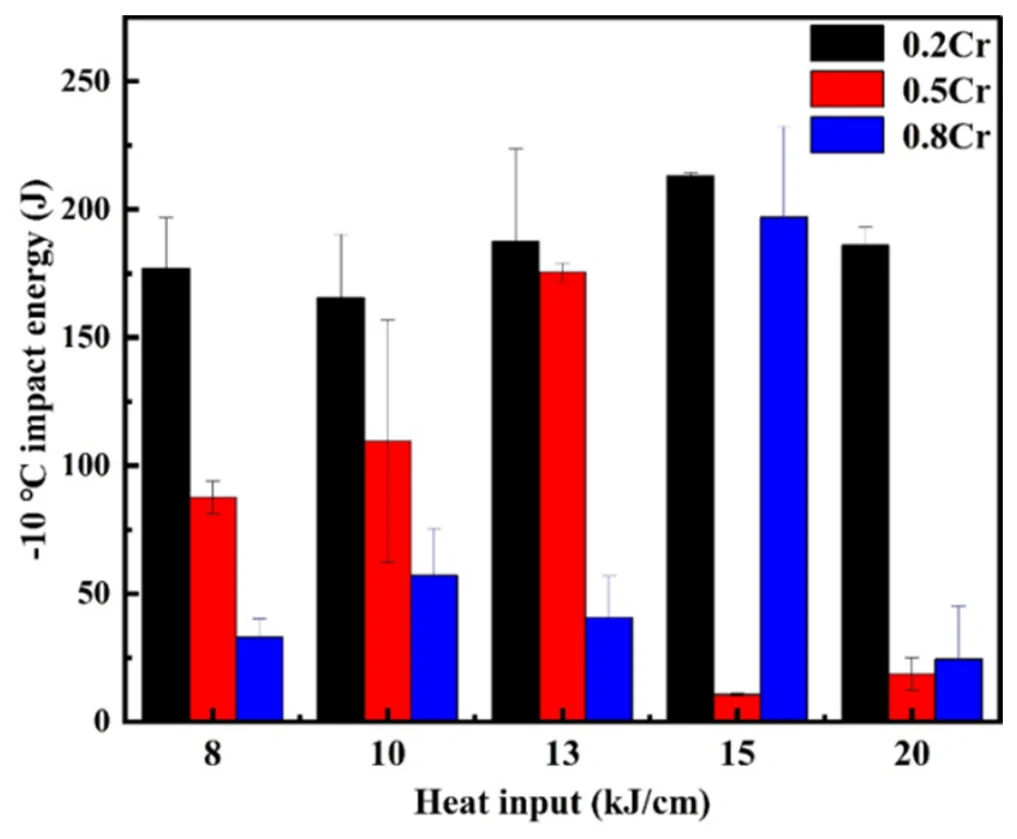
Low temperature impact energy of CGHAZ at −10 °C with different welding heat inputs.

Figure 3 illustrates the correlation between welding heat input and −10 °C impact energy of the CGHAZ for the 0.2Cr, 0.5Cr and 0.8Cr experimental steels. The impact toughness of the heat-affected zone in pipe welding for this study’s supported project shall not be less than 50 J at −10 °C. From the histogram data in Figure 3, the range of valid welding heat input for maintaining satisfactory CGHAZ impact toughness gradually narrows with increasing Cr mass fraction of the steel. Specifically, 0.2Cr steel maintains favorable toughness over the entire range of heat input from 8 to 20 kJ/cm. The high-toughness heat input interval for 0.5Cr steel is limited to 8–13 kJ/cm. It is only with a heat input of 15 kJ/cm that 0.8Cr steel can achieve relatively high toughness. Existing studies have proven that welding heat input exerts a remarkable influence on the low-temperature toughness of CGHAZ in pipeline steel. Excessively high heat input readily induces abnormal grain growth and consequently toughness degradation. On the contrary, too low heat input leads to an extremely fast cooling rate during welding, which promotes the formation of abundant hard and brittle non-equilibrium phases such as martensite and bainite inside the CGHAZ and brings about a pronounced hardening effect, thereby deteriorating the low-temperature impact resistance of welded joints likewise. A moderate and matched heat input parameter enables precise regulation of the welding thermal cycle. It not only avoids excessive grain coarsening but also suppresses the generation of brittle hardened microstructures, facilitating the formation of fine and homogeneous carbide-free bainite microstructures (such as bainite ferrite or granular bainite) within the CGHAZ. Such microstructures possess outstanding crack arresting capacity and plastic deformability, which greatly improves the low-temperature toughness and service stability of pipeline steel welded joints [25]. Therefore, the effect of different heat inputs on the microstructure of the experimental steel will be further investigated.

**Figure 4:**
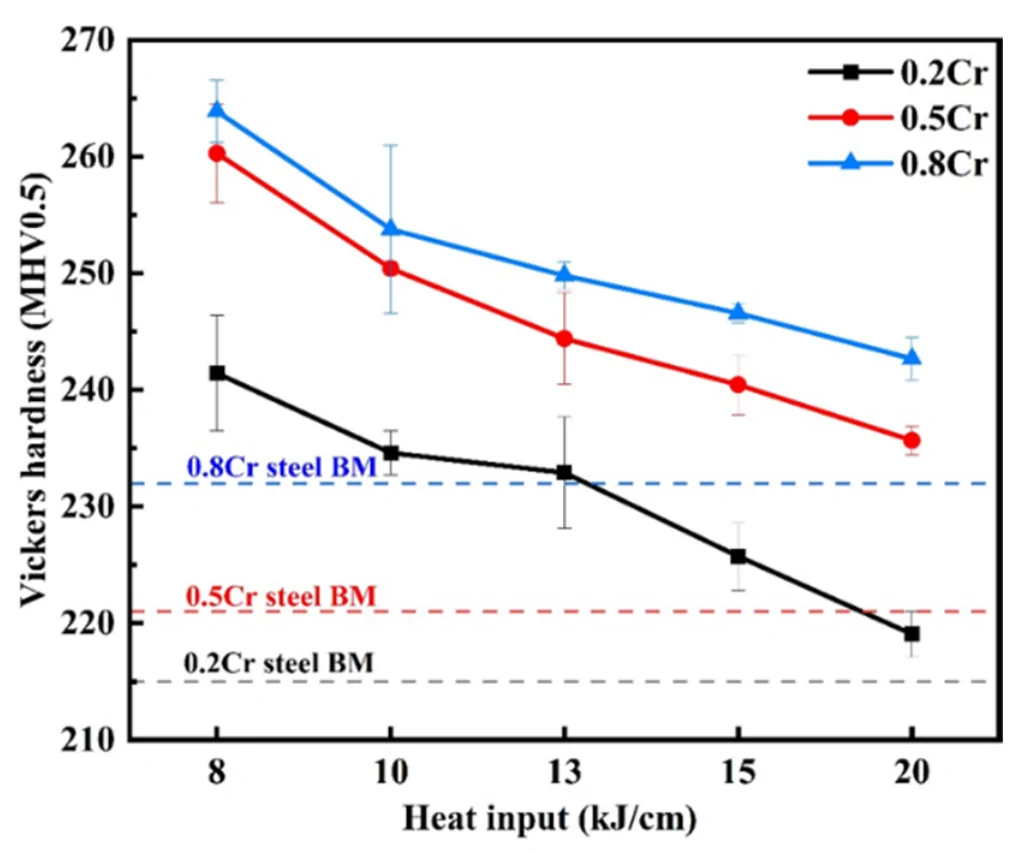
Microhardness of CGHAZ with different welding heat input (HV0.5).

Figure 4 depicts the relationship between microhardness and welding heat input of CGHAZ in the 0.2Cr, 0.5Cr and 0.8Cr experimental steels, where the dashed line denotes the hardness of base metal (BM). It is observed that the microhardness of CGHAZ for all three steels decreases monotonically with the increase in welding heat input. At identical heat input levels, the CGHAZ microhardness rises remarkably as Cr content increases. At the low heat input of 8 kJ/cm, the CGHAZ microhardness of each steel is distinctly higher than that of its corresponding base metal, with the 0.8Cr steel exhibiting the maximum CGHAZ hardness. As the welding heat input rises continuously, the welding cooling rate reduces, leading to a steady drop in CGHAZ hardness. At the high heat input of 20 kJ/cm, the CGHAZ hardness of the 0.2Cr steel falls below its base metal hardness. By contrast, although the CGHAZ hardness values of the 0.5Cr and 0.8Cr experimental steels decline simultaneously, they remain higher than their respective base metal benchmarks throughout the tested range.

### 3.3. Effect of Welding Heat Input on Microstructure Evolution of CGHAZ

**Figure 5:**
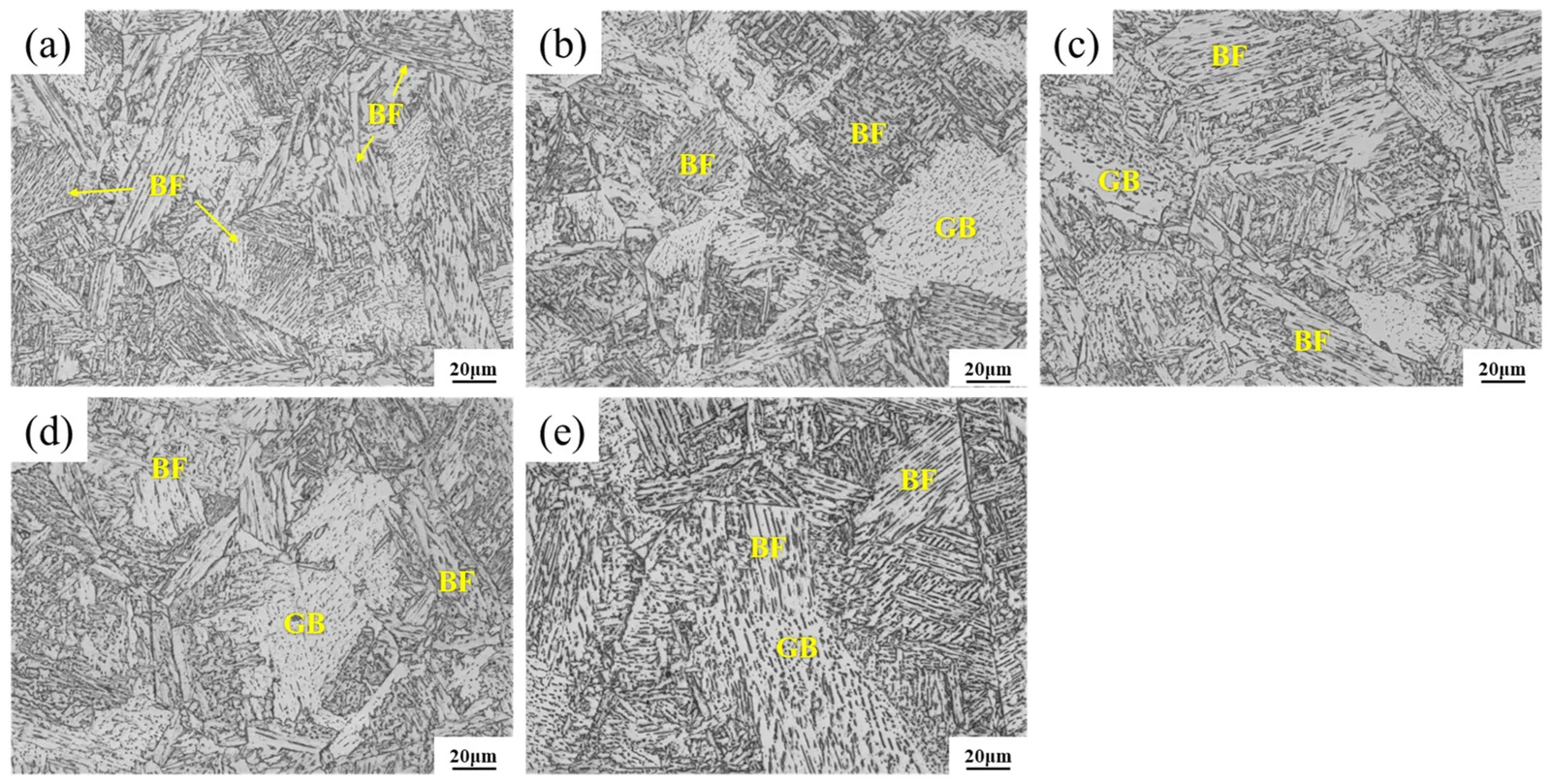
Metallographic images of CGHAZ microstructure of 0.2Cr experimental steel under different welding heat inputs: (**a**) 8 kJ/cm; (**b**) 10 kJ/cm; (**c**) 13 kJ/cm; (**d**) 15 kJ/cm; (**e**) 20 kJ/cm.

**Figure 6:**
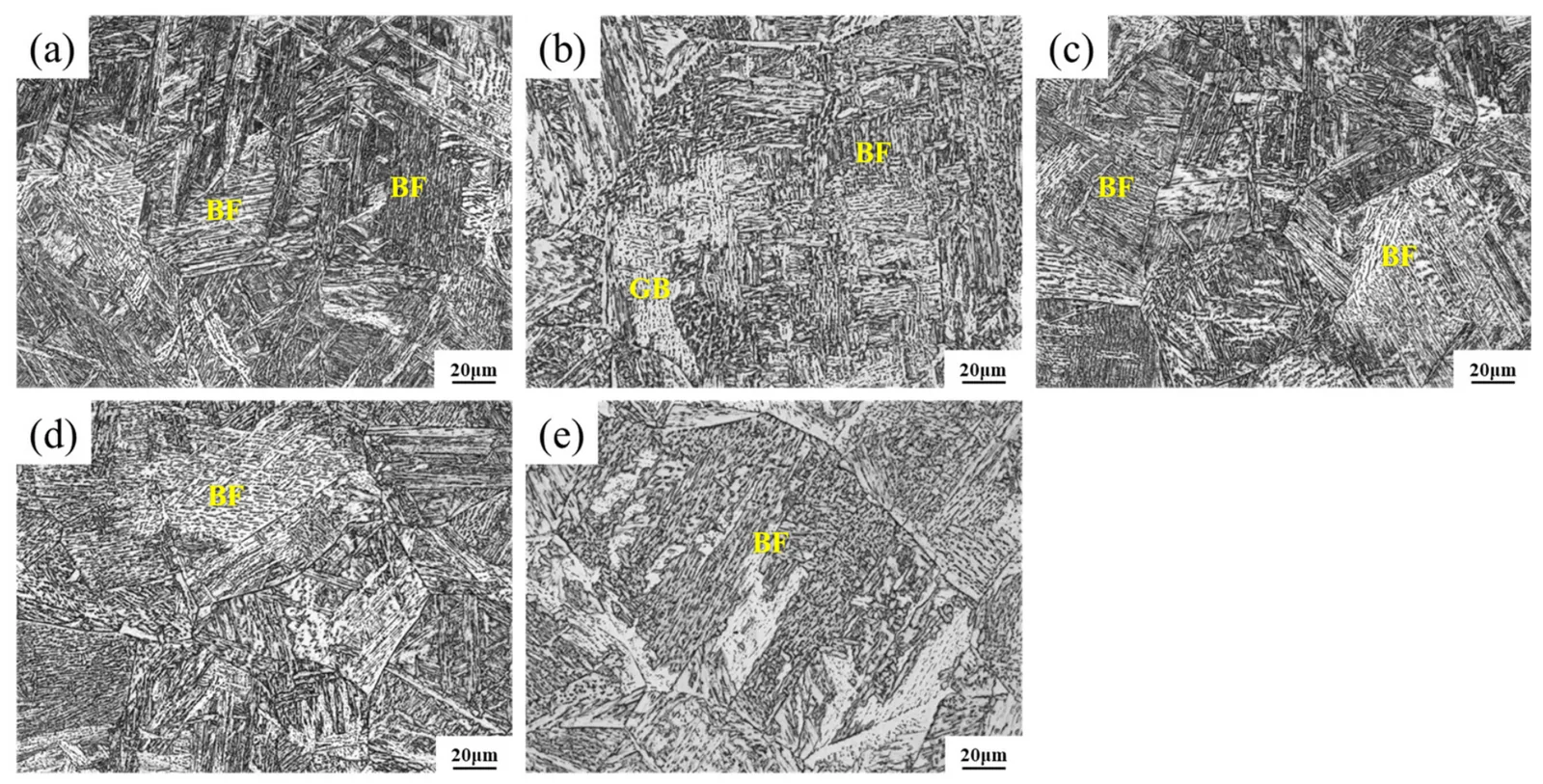
Metallographic images of CGHAZ microstructure of 0.5Cr experimental steel under different welding heat inputs: (**a**) 8 kJ/cm; (**b**) 10 kJ/cm; (**c**) 13 kJ/cm; (**d**) 15 kJ/cm; (**e**) 20 kJ/cm.

**Figure 7:**
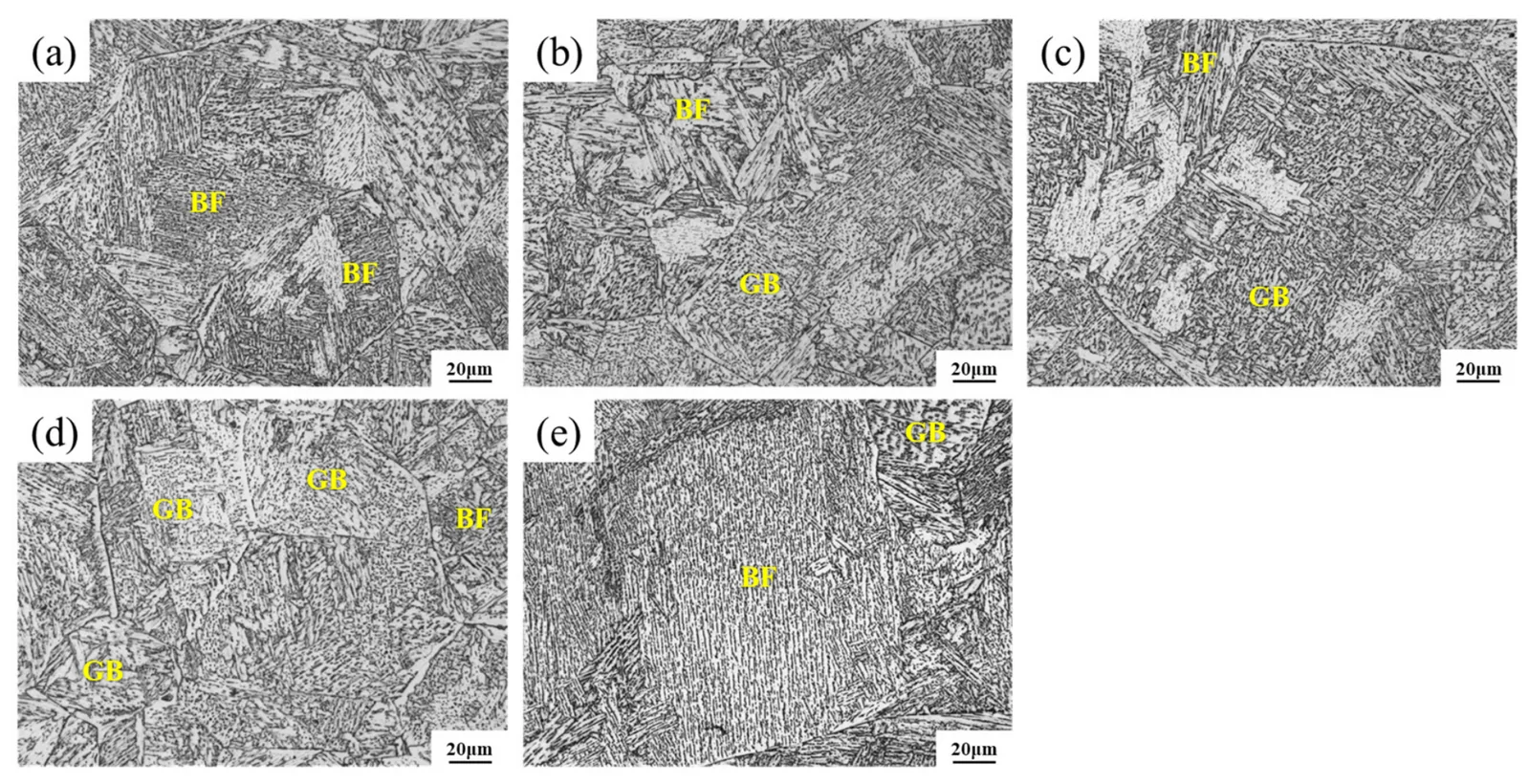
Metallographic images of CGHAZ microstructure of 0.8Cr experimental steel under different welding heat inputs: (**a**) 8 kJ/cm; (**b**) 10 kJ/cm; (**c**) 13 kJ/cm; (**d**) 15 kJ/cm; (**e**) 20 kJ/cm.

Figure 5, Figure 6 and Figure 7 display the optical micrographs of CGHAZ for the three experimental steels under various welding heat inputs, respectively. The prior austenite grain size increases significantly with rising welding heat input for a fixed Cr content. At low heat inputs (8–10 kJ/cm), the CGHAZ microstructure of all three steels is predominantly composed of bainitic ferrite with fine lath bundles. As the heat input gradually increases to 13–20 kJ/cm, the volume fraction of bainitic ferrite declines, accompanied by a corresponding increase in the fraction of granular bainite.

**Figure 8:**
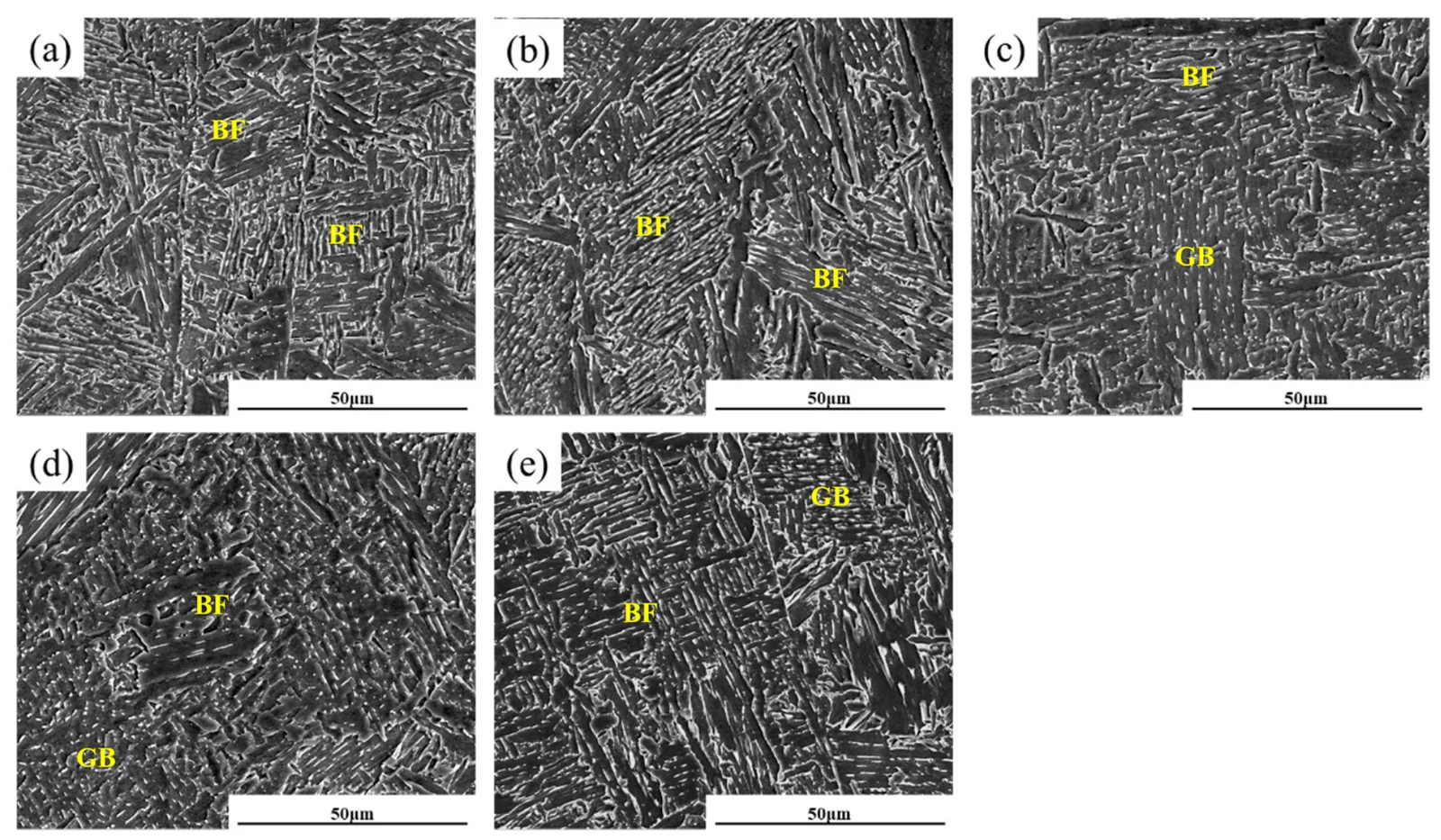
SEM image of CGHAZ microstructure of 0.2Cr experimental steel under different welding heat input: (**a**) 8 kJ/cm; (**b**) 10 kJ/cm; (**c**) 13 kJ/cm; (**d**) 15 kJ/cm; (**e**) 20 kJ/cm.

**Figure 9:**
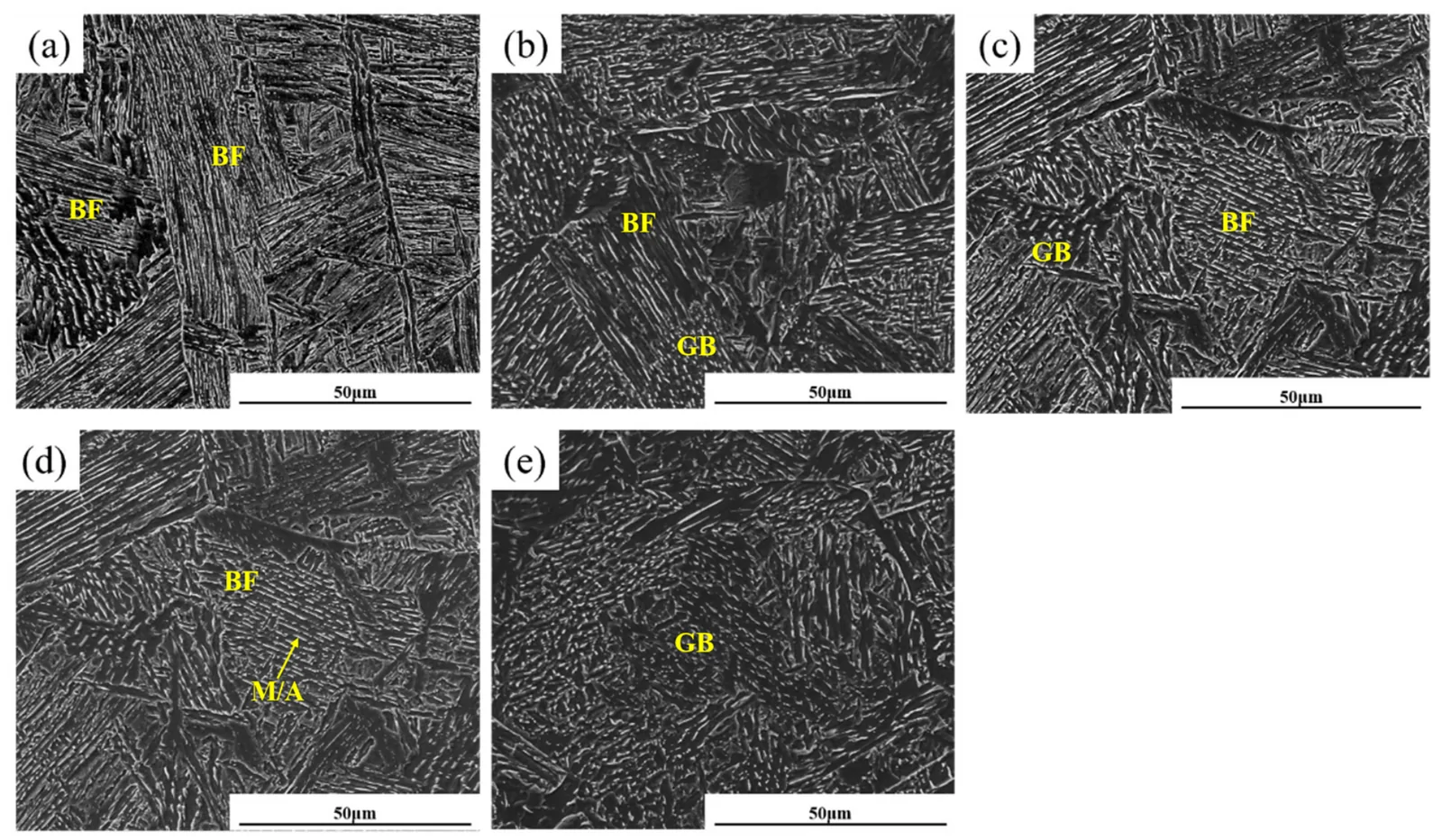
SEM image of CGHAZ microstructure of 0.5Cr experimental steel under different welding heat input: (**a**) 8 kJ/cm; (**b**) 10 kJ/cm; (**c**) 13 kJ/cm; (**d**) 15 kJ/cm; (**e**) 20 kJ/cm.

**Figure 10:**
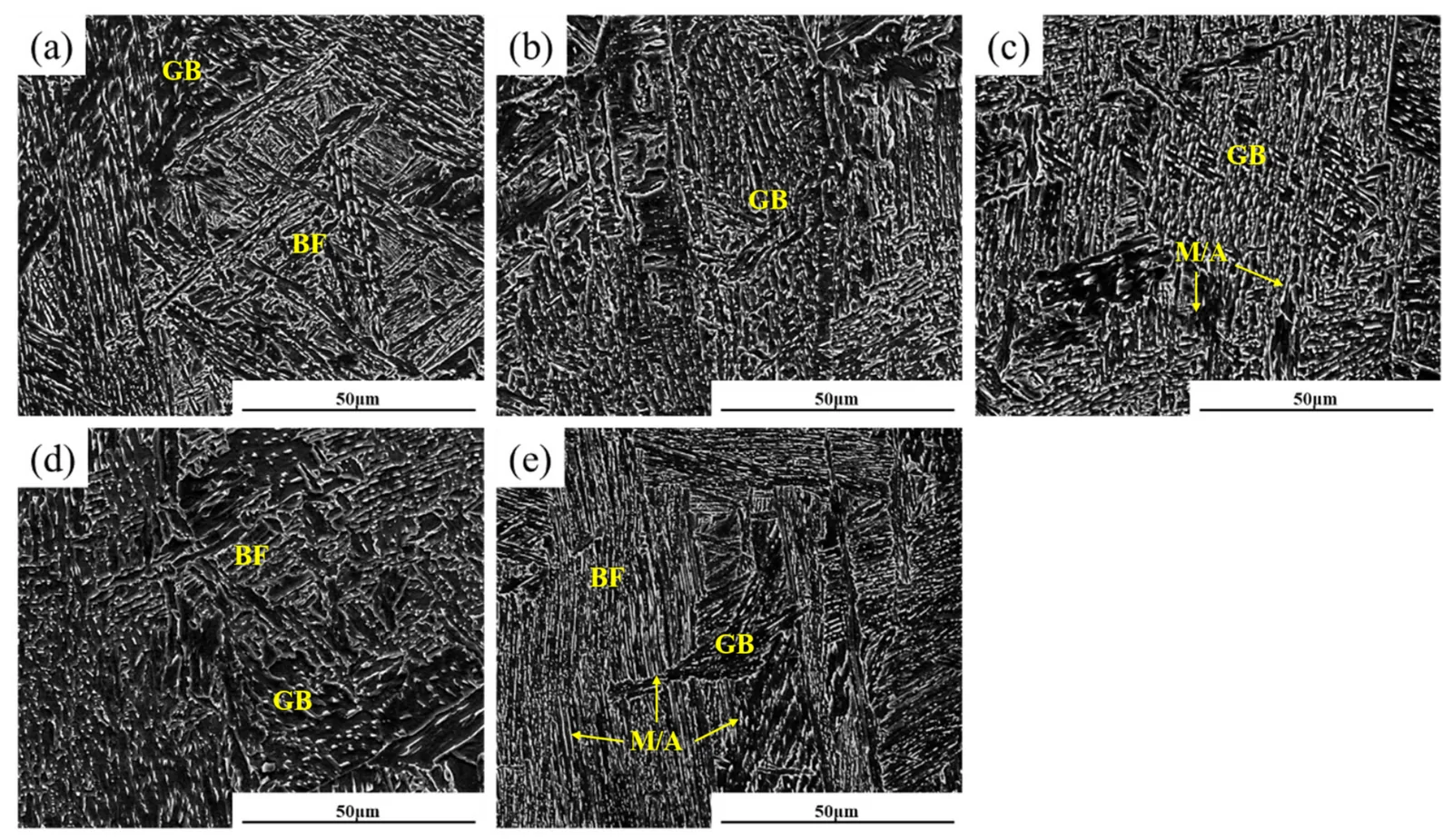
SEM image of CGHAZ microstructure of 0.8Cr experimental steel under different welding heat inputs: (**a**) 8 kJ/cm; (**b**) 10 kJ/cm; (**c**) 13 kJ/cm; (**d**) 15 kJ/cm; (**e**) 20 kJ/cm.

Figure 8, Figure 9 and Figure 10 present the SEM micrographs of CGHAZ in the 0.2Cr, 0.5Cr and 0.8Cr experimental steels under different welding heat inputs (8, 10, 13, 15, 20 kJ/cm), respectively. Under the contrast of secondary electron images, the interlaced lath bundle structures inside bainitic ferrite (BF) and the morphology of granular M/A constituents distributed in granular bainite (GB) can be clearly observed. With the rise in heat input, all three experimental steels follow the same microstructure evolution law: the BF lath bundles broaden, granular bainite forms partially, and the quantity of M/A constituents within GB rises. Nevertheless, the variation degree differs remarkably with increasing Cr content. For the 0.2Cr steel, the broadening degree of BF lath bundles is limited, and the M/A constituents inside GB remain fine. Consequently, this steel maintains relatively high toughness across the entire tested heat input range. For the 0.5Cr steel, elevated Cr content improves hardenability. The well-developed lath structure of BF contributes to toughness improvement, yet the accompanying increased phase transformation stress exerts an adverse effect on toughness. Under the coupled effect of these two factors, its overall toughness is inferior to that of the 0.2Cr steel. When the heat input increases to 15–20 kJ/cm, massive generation of GB brings about a sharp rise in the fraction of M/A constituents, triggering a drastic drop in toughness. As for the 0.8Cr steel, its hardenability is further enhanced, accompanied by a higher volume fraction of M/A constituents in the microstructure, as well as an increased martensite proportion inside the M/A constituents, which ultimately leads to severe toughness deterioration.

**Figure 11:**
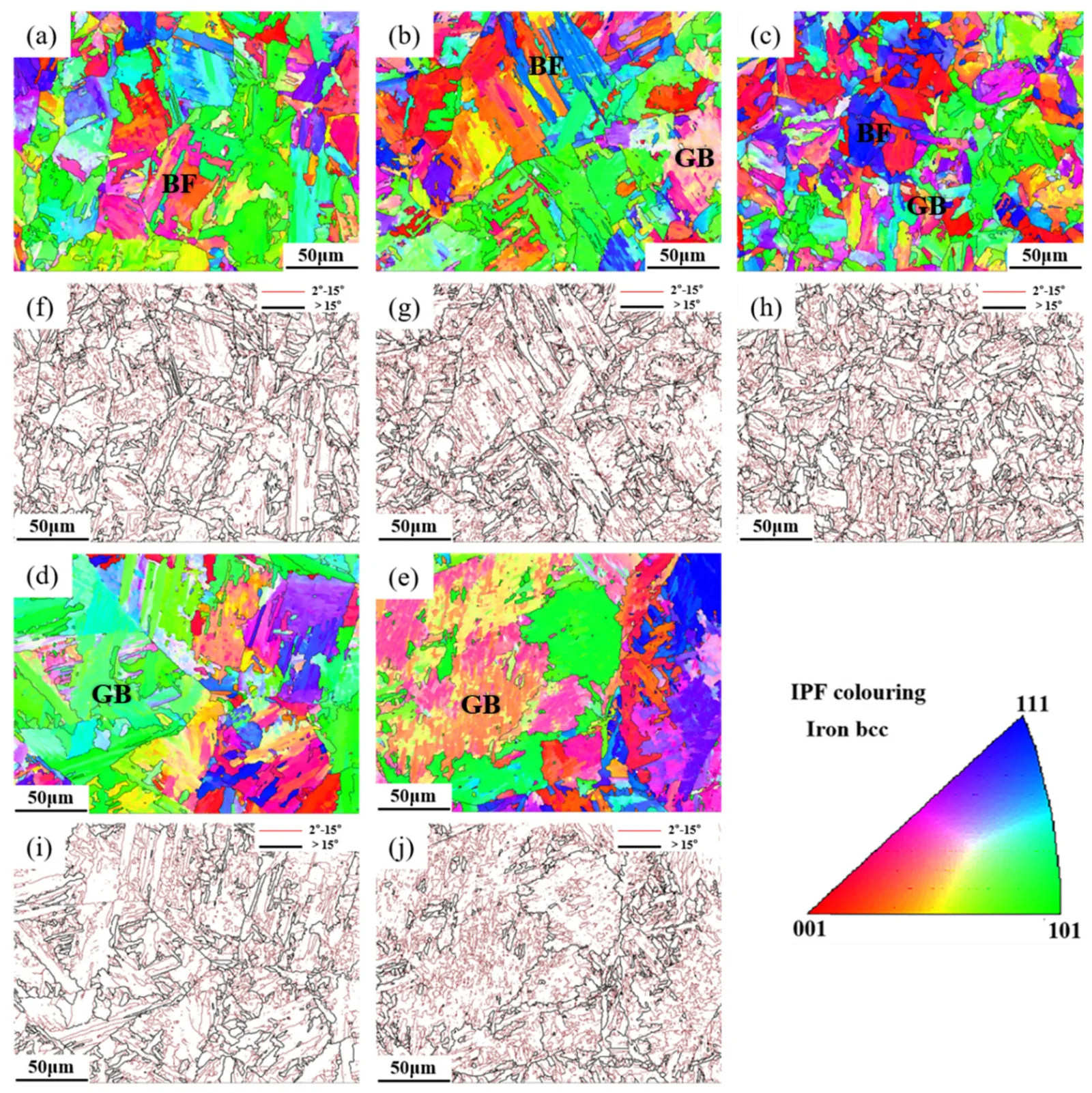
EBSD characterization of 0.5Cr experimental steel under different heat inputs: (**a**,**f**) 8 kJ/cm, (**b**,**g**) 10 kJ/cm; (**c**,**h**) 13 kJ/cm; (**d**,**i**) 15 kJ/cm; (**e**,**j**) 20 kJ/cm.

**Figure 12:**
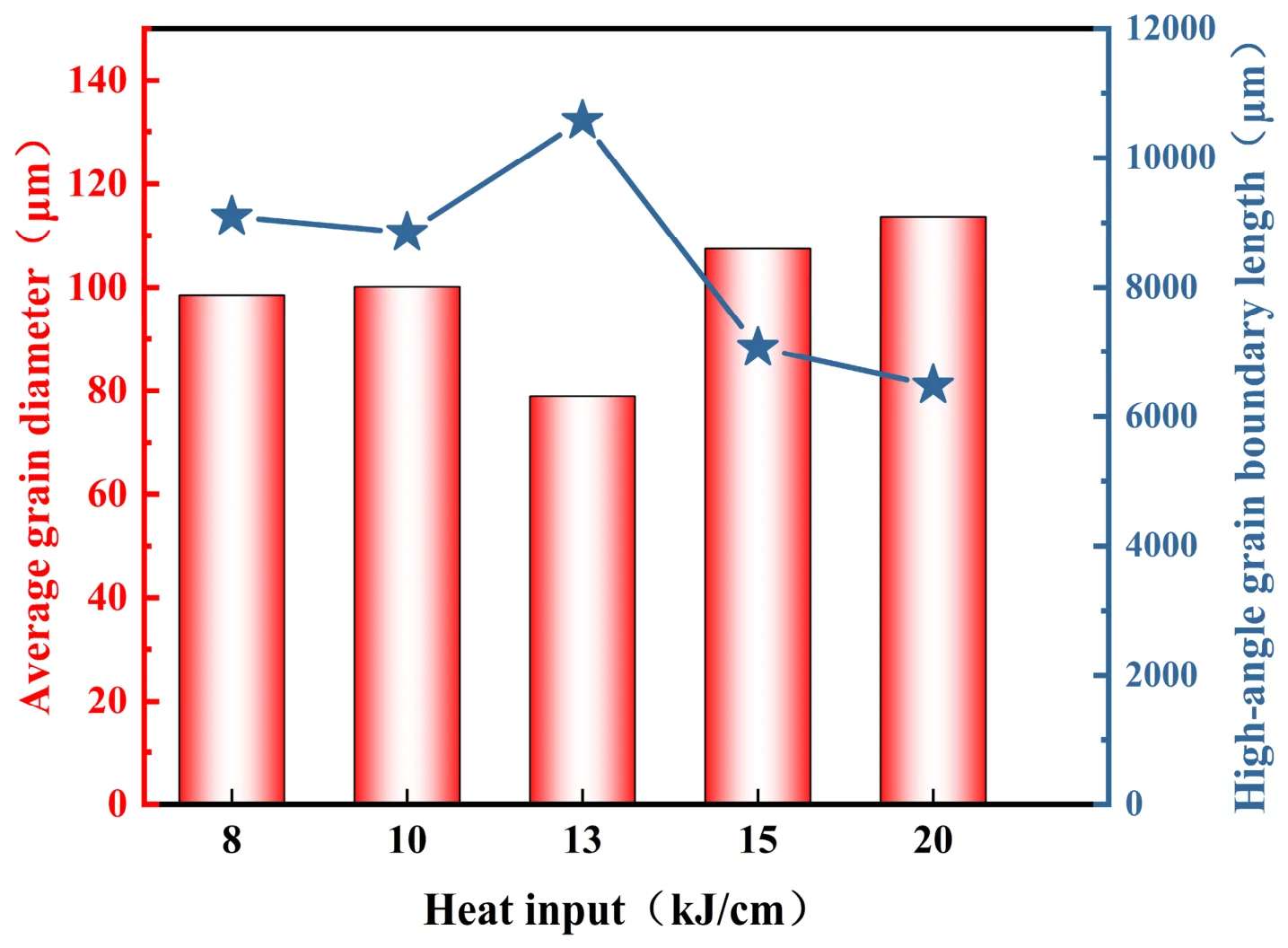
Statistical diagram of average grain area and large-angle grain boundary length of 0.5Cr experimental steel.

EBSD characterization was performed on the 0.5Cr experimental steel to further investigate the effect of welding heat input on the microstructure and grain evolution of CGHAZ. As shown in the EBSD results in Figure 11, the prior austenite grain size increases significantly with the elevation of welding heat input. Quantitative statistical measurements based on the linear intercept method from the grain boundary maps (Figure 11f–j) further verify this variation trend. The average prior austenite grain sizes are quantitatively determined as 28.6 μm, 37.2 μm, 45.9 μm, 56.3 μm, and 69.7 μm under heat inputs of 8, 10, 13, 15, and 20 kJ/cm, respectively. Visually, the prior austenite grain boundaries of the specimen treated at 20 kJ/cm (Figure 11e,j) are considerably wider and coarser than those of the 8 kJ/cm specimen (Figure 11a,f), confirming the continuous coarsening of prior austenite grains with increasing heat input based on numerical data rather than qualitative description alone. Figure 11 displays the inverse pole figure (IPF) and high/low angle grain boundary maps of 0.5Cr steel under different heat input conditions, which further reveal the microstructure evolution characteristics. At low heat inputs (8–10 kJ/cm), the CGHAZ microstructure is predominantly composed of bainitic ferrite (BF). At the intermediate heat input of 13 kJ/cm, the microstructure consists of a mixed structure of BF and fine granular bainite (GB). In this case, the fine granular bainite contains small ferrite matrix grains with irregular grain boundaries, which significantly refines the effective grain size. Herein, the effective grain is defined as the grain enclosed by high-angle grain boundaries with a misorientation angle greater than 15° between adjacent grains. Under high heat inputs (15–20 kJ/cm), the microstructure is mainly dominated by granular bainite accompanied by prominent grain coarsening. In the coarsened GB regions, internal subgrains are mostly bounded by low-angle grain boundaries, resulting in a remarkable increase in the overall effective grain size. The statistically measured effective grain sizes and high-angle grain boundary lengths under various heat inputs are summarized in Figure 12. Combined with the previous low-temperature toughness test results, it can be concluded that the variation in effective grain size induced by different welding heat inputs is one of the dominant factors determining the low-temperature toughness of the CGHAZ in 0.5Cr experimental steel.

We have previously performed a gradient thermal simulation study on X80 pipe steel with Cr content ranging from 0 to 0.4 wt% and showed that Cr enhances austenite hardenability, promotes granular bainite conversion, and increases the volume fraction of M/A constituents in the microstructure. In addition, the study indicated that the nanohardness of the M/A constituent increased from 5.74–6.74 GPa to 6.31–8.01 GPa with an increase in Cr content from 0.1 to 0.4 wt%. The high-hardness M/A constituent is prone to initiate micro-cracks, leading to a significant reduction in the impact toughness at Cr content up to 0.4 wt%, consistent with the overall trend observed in this study [26]. Wang et al. [27] investigated the effect of heat input on the microstructure and properties of X100 pipeline steel in the coarse grain heat-affected zone (CGHAZ). When the heat input is less than 8 kJ/cm, the microstructure consists of lath martensite. The microstructure transforms into granular bainite when the heat input ranges from 26 to 36 kJ/cm. Heat input has little effect on the hardness, but significantly affects the impact toughness. As heat input increased, the overall content of M/A constituents increased, while the number of M/A constituents smaller than 1 μm decreased. Moreover, when the heat input exceeds 15 kJ/cm, the M/A constituent forms a necklace-like distribution along the original austenite grain boundaries, leading to a significant reduction in toughness. Zhang et al. [28] reviewed the relationship between alloying elements and bainite transformations in high-strength steel welds, noting that the primary role of Cr in welds is to promote the formation of GB and M/A constituents, as well as their microstructural evolution.

## 4. Discussion

### 4.1. Study on the Effect of Welding Heat Input on Impact Fracture Behavior of CGHAZ

**Figure 13:**
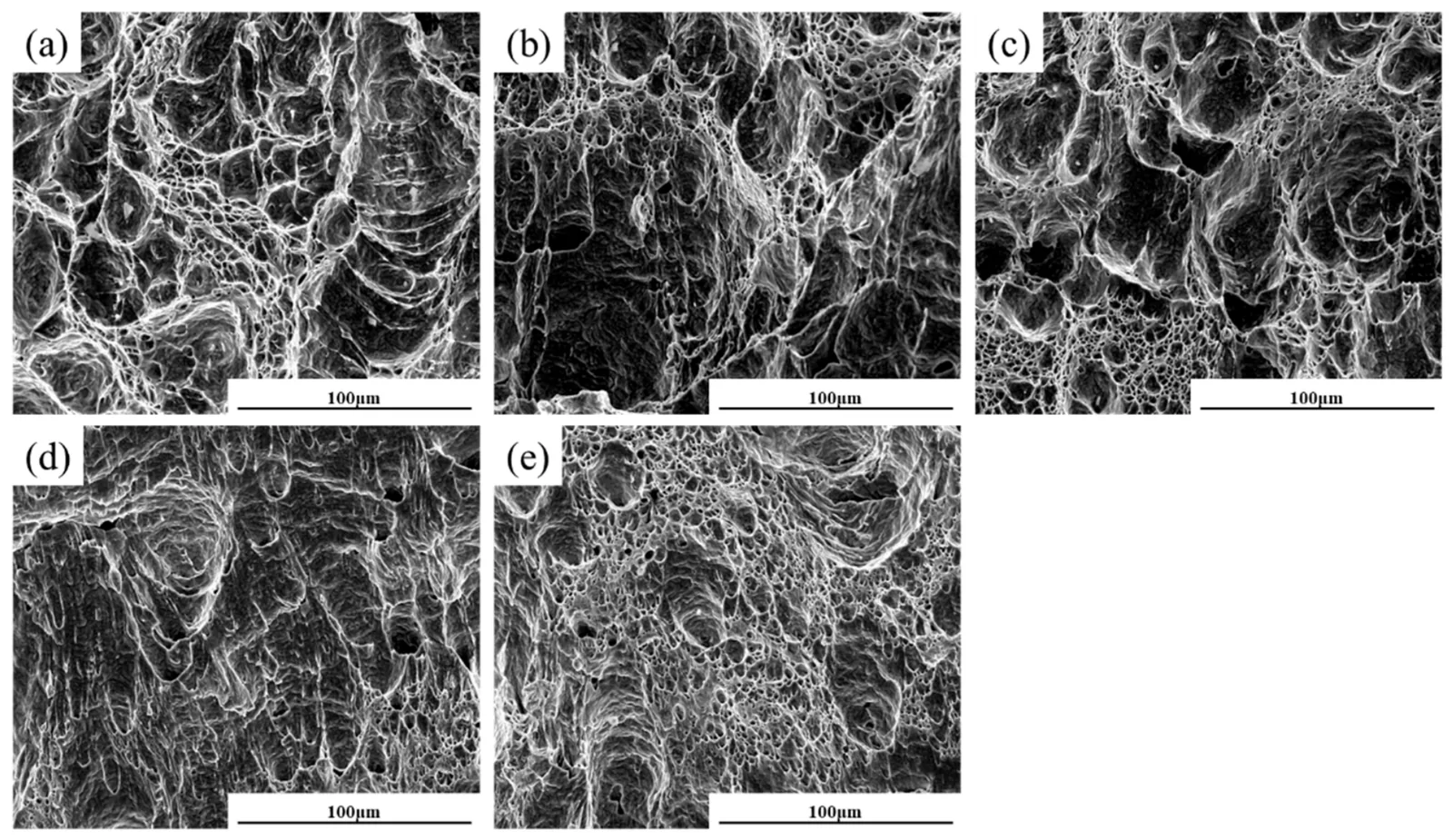
SEM morphology of impact fracture of CGHAZ under different welding heat inputs of 0.2Cr experimental steel: (**a**) 8 kJ/cm, (**b**) 10 kJ/cm, (**c**) 13 kJ/cm, (**d**) 15 kJ/cm, (**e**) 20 kJ/cm.

**Figure 14:**
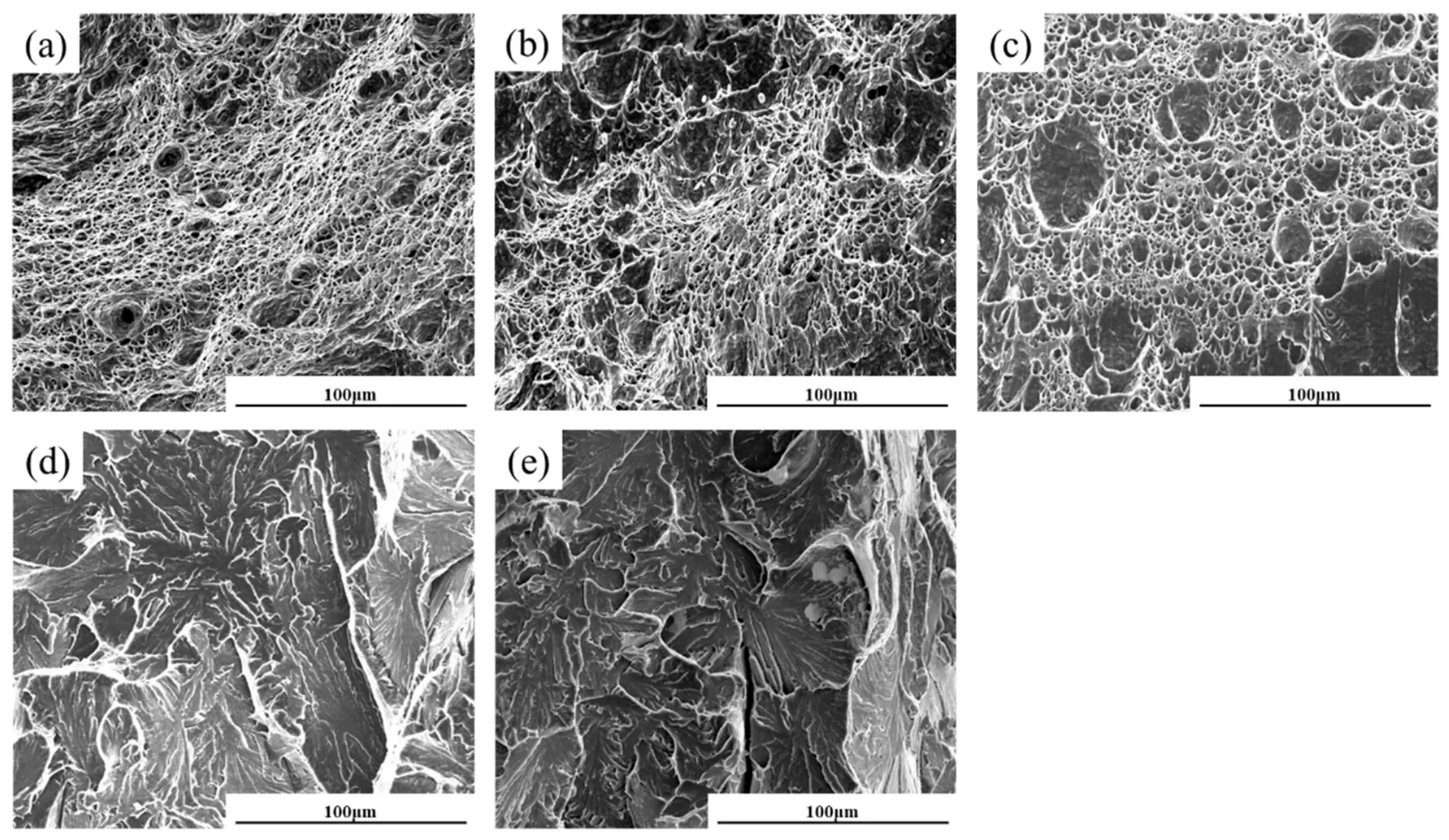
SEM morphology of impact fracture of CGHAZ under different welding heat inputs of 0.5Cr experimental steel: (**a**) 8 kJ/cm, (**b**) 10 kJ/cm, (**c**) 13 kJ/cm, (**d**) 15 kJ/cm, (**e**) 20 kJ/cm.

**Figure 15:**
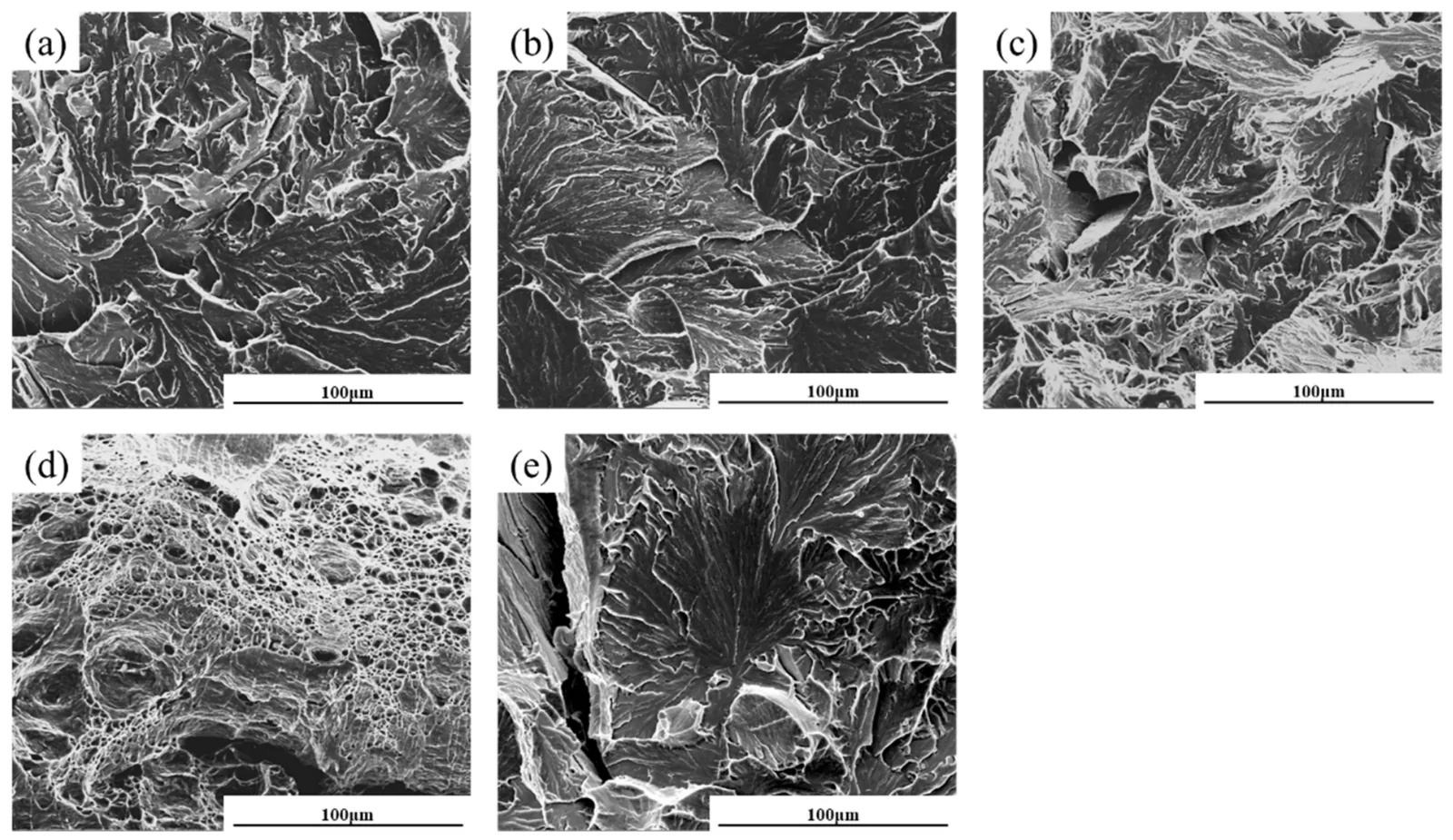
SEM morphology of impact fracture of CGHAZ under different welding heat inputs of 0.8Cr experimental steel: (**a**) 8 kJ/cm, (**b**) 10 kJ/cm, (**c**) 13 kJ/cm, (**d**) 15 kJ/cm, (**e**) 20 kJ/cm.

The SEM fractographs of CGHAZ for the 0.2Cr, 0.5Cr and 0.8Cr experimental steels under different welding heat inputs are presented in Figure 13, Figure 14 and Figure 15. Dimple features dominate the fracture surfaces for the 0.2Cr steel over the entire heat input range of 8–20 kJ/cm, the 0.5Cr steel at 8 kJ/cm, 10 kJ/cm and 13 kJ/cm, as well as the 0.8Cr steel at 15 kJ/cm, as displayed in Figure 13a–e, Figure 14a–c and Figure 15d. Such morphology can prolong the ductile crack propagation stage and elevate the energy absorbed during crack growth. Existing research has confirmed that the size and depth of dimples are directly correlated with material toughness. High-toughness materials usually exhibit large and deep dimples on fracture surfaces, whereas small and shallow dimples correspond to relatively poor toughness [29]. The fracture morphology evolution under the above conditions agrees well with the measured impact toughness data. For the 0.5Cr steel tested at heat inputs of 8 kJ/cm, 10 kJ/cm, 15 kJ/cm and 20 kJ/cm, tear ridges can be observed at the junctions of cleavage facets, showing typical quasi-cleavage fracture characteristics. When the 0.8Cr steel is subjected to 8 kJ/cm, 10 kJ/cm, 13 kJ/cm and 20 kJ/cm heat inputs, obvious river-like patterns appear on the fracture surfaces; inclusions, voids and other defects are also visible on cleavage planes, which are typical features of cleavage fracture and correspond to low impact toughness.

**Figure 16:**
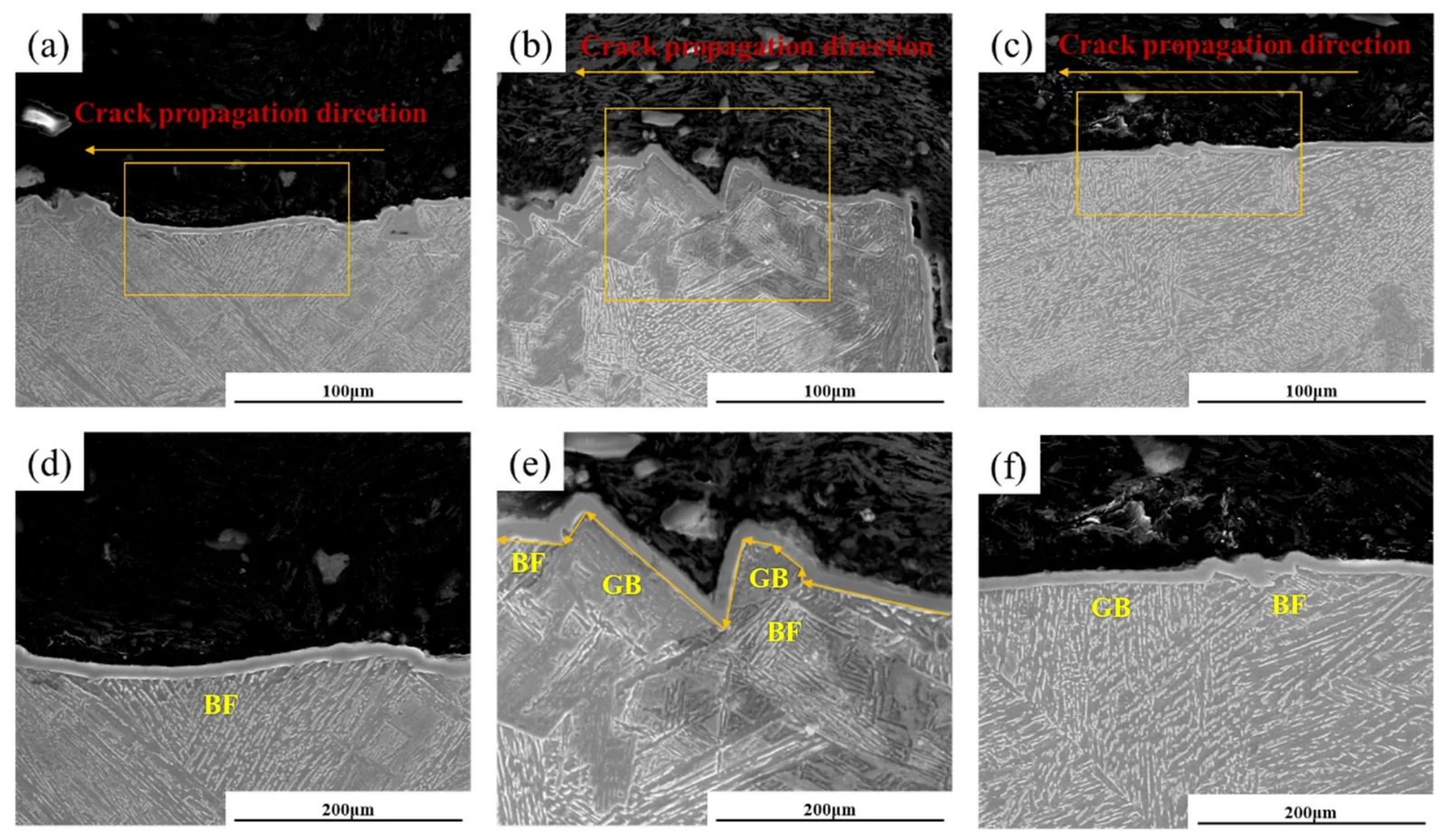
Main crack propagation path in brittle zone of CGHAZ impact fracture of 0.5Cr experimental steel under different welding heat inputs: (**a**) 8 kJ/cm; (**b**) 13 kJ/cm; (**c**) 15 kJ/cm; (**d**) 8 kJ/cm; (**e**) 13 kJ/cm; (**f**) 15 kJ/cm.

The impact fracture surfaces of CGHAZ in the 0.5Cr steel at welding heat inputs of 8 kJ/cm, 13 kJ/cm and 15 kJ/cm were selected to characterize the propagation direction of main cracks in the brittle zone, and the results are presented in Figure 16. Minor microstructure deformation can be observed in all brittle zones. The crack paths are relatively straight at heat inputs of 8 kJ/cm and 15 kJ/cm, while they become tortuous at 13 kJ/cm. A more tortuous crack propagation path implies stronger resistance encountered during crack growth and higher energy consumption, corresponding to larger impact absorbed energy of the material, which is consistent with the low-temperature toughness test results.

At low heat input, a high cooling rate promotes the formation of abundant bainitic ferrite in the steel. As shown in Figure 16a,d, cracks propagate straightly inside bainitic ferrite. The elongated M/A constituents within bainitic ferrite barely hinder crack growth. Particularly when the crack propagation direction is parallel to these elongated M/A constituents, few obstacles exist along the crack path, and less energy is required for crack extension. Once microcracks initiate, unstable crack propagation is likely to occur, leading to a sharp deterioration in material toughness [30,31]. When the heat input rises to 13 kJ/cm, Figure 16b,e reveal that cracks propagate linearly within granular bainite and bainitic ferrite, yet undergo large-angle deflection upon encountering the grain boundaries outside bainitic ferrite. This process consumes substantial energy and renders the crack path much more tortuous [32,33]. With a further increase in welding heat input up to 15 kJ/cm, the crack path becomes nearly straight, as displayed in Figure 16c,f. The microstructure at this condition consists of coarse granular bainite with a high-volume fraction of internal M/A constituents, which exert a limited blocking effect on crack growth and consequently reduce the material toughness [34].

**Figure 17:**
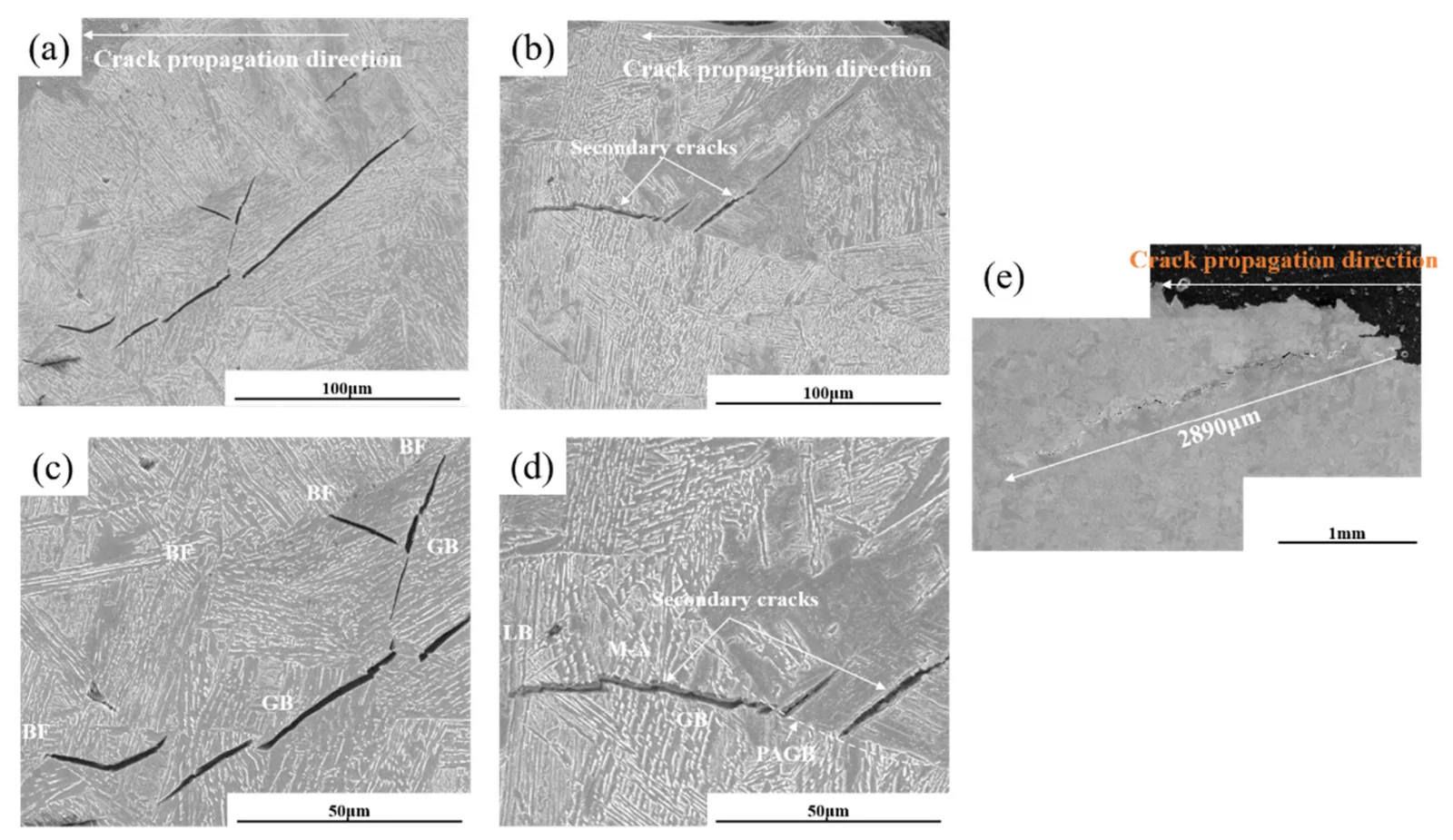
Morphology of secondary cracks in part of 0.5Cr experimental steel: (**a**,**c**) 8 kJ/cm; (**b**,**d**,**e**) 15 kJ/cm.

**Figure 18:**
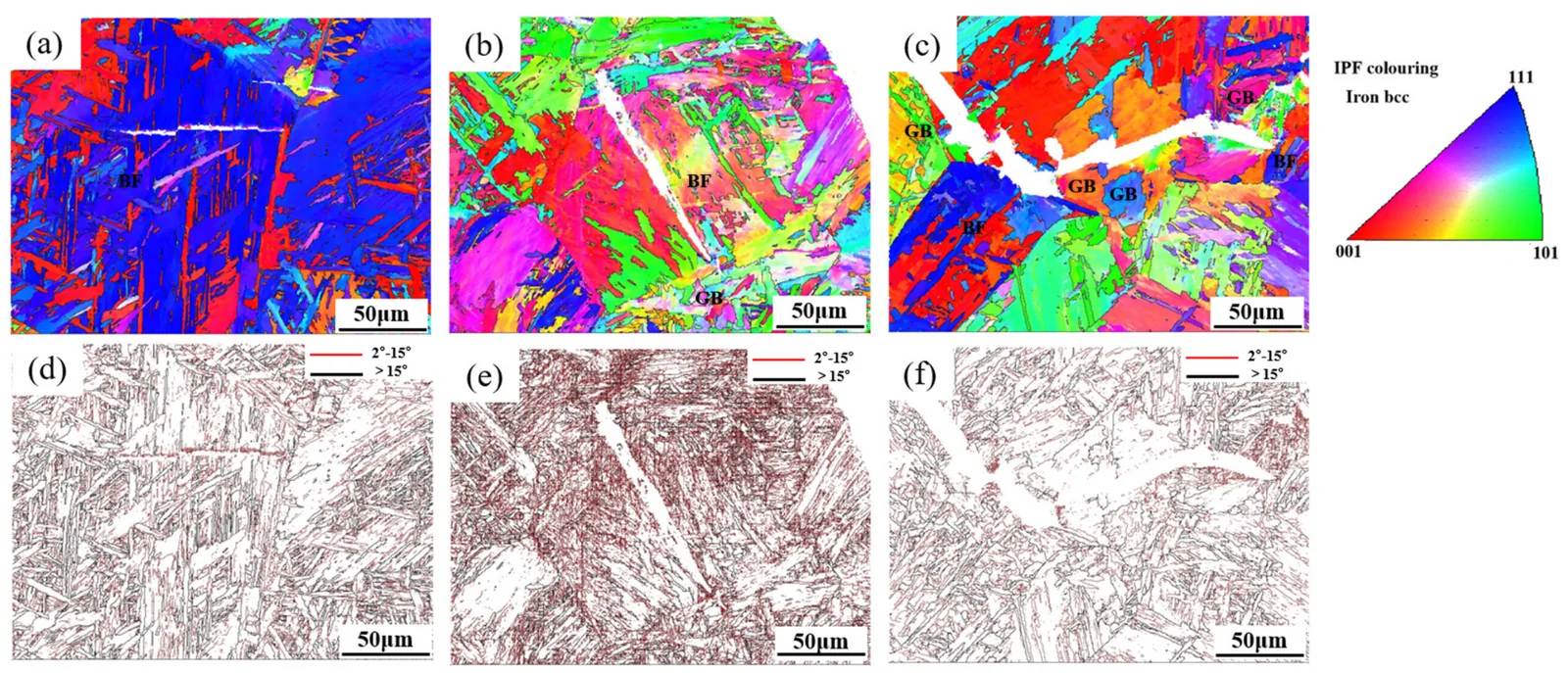
EBSD images of secondary crack propagation path of 0.5Cr experimental steel: (**a**–**c**) grain boundary misorientation map; (**d**–**f**) inverse pole figure.

Secondary cracks refer to newly generated cracks initiated adjacent to primary cracks in materials or structures, induced by stress concentration, fatigue, corrosion, material defects and other factors. They generally emerge at or near the tip of the primary crack, and their propagation directions can be parallel or inclined at a certain angle to the primary crack, with diverse morphologies such as linear, curved and branched shapes. Figure 17 shows the typical morphology of partial secondary cracks in the 0.5Cr experimental steel. A large number of secondary cracks are distributed around the main crack under low heat input (8 kJ/cm) and relatively high heat input (15 kJ/cm). As displayed in Figure 17e, long disconnected cracks are also detected at the heat input of 15 kJ/cm. Magnified observation of selected cracks in Figure 18c,d indicates that secondary cracks tend to nucleate at M/A constituents with specific geometries. After initiation, secondary cracks readily propagate inside granular bainite with barely any resistance. When the propagating secondary cracks encounter the grain boundaries of bainitic ferrite or prior austenite grain boundaries, they deflect or even arrest completely [35].

Figure 18 presents EBSD maps showing the propagation paths of secondary cracks in the 0.5Cr experimental steel. It can be observed that the interfaces between lath bundles inside bainitic ferrite (BF) are mostly high-angle grain boundaries (HAGBs). Most laths are arranged in parallel but vary in size, while a small fraction of laths interlock with neighboring ones (Figure 18a,b). Secondary cracks propagating within BF tend to be arrested at lath boundaries with large misorientation angles. In contrast, granular bainite (GB) is dominated by low-angle grain boundaries (LAGBs), which impose nearly no blocking effect on crack growth, and the average grain size of granular bainite in Figure 18d is measured to be 4.38 μm (Figure 18d).

Notably, when cracks propagate among fine GB grains with average sizes of 4.8 μm and 5.72 μm for Figure 18e,f, respectively, they undergo sharp deflection at the high-angle boundaries separating adjacent GB grains. This process consumes substantial energy and thereby remarkably improves the low-temperature toughness (Figure 18e,f). Intersecting high-angle grain boundaries oriented in diverse directions deliver the strongest resistance to crack extension. This serves as one of the reasons why the impact toughness of the 0.5Cr and 0.8Cr steels first rises and then falls within the tested heat input range of 8–20 kJ/cm^2^ [36].

**Figure 19:**
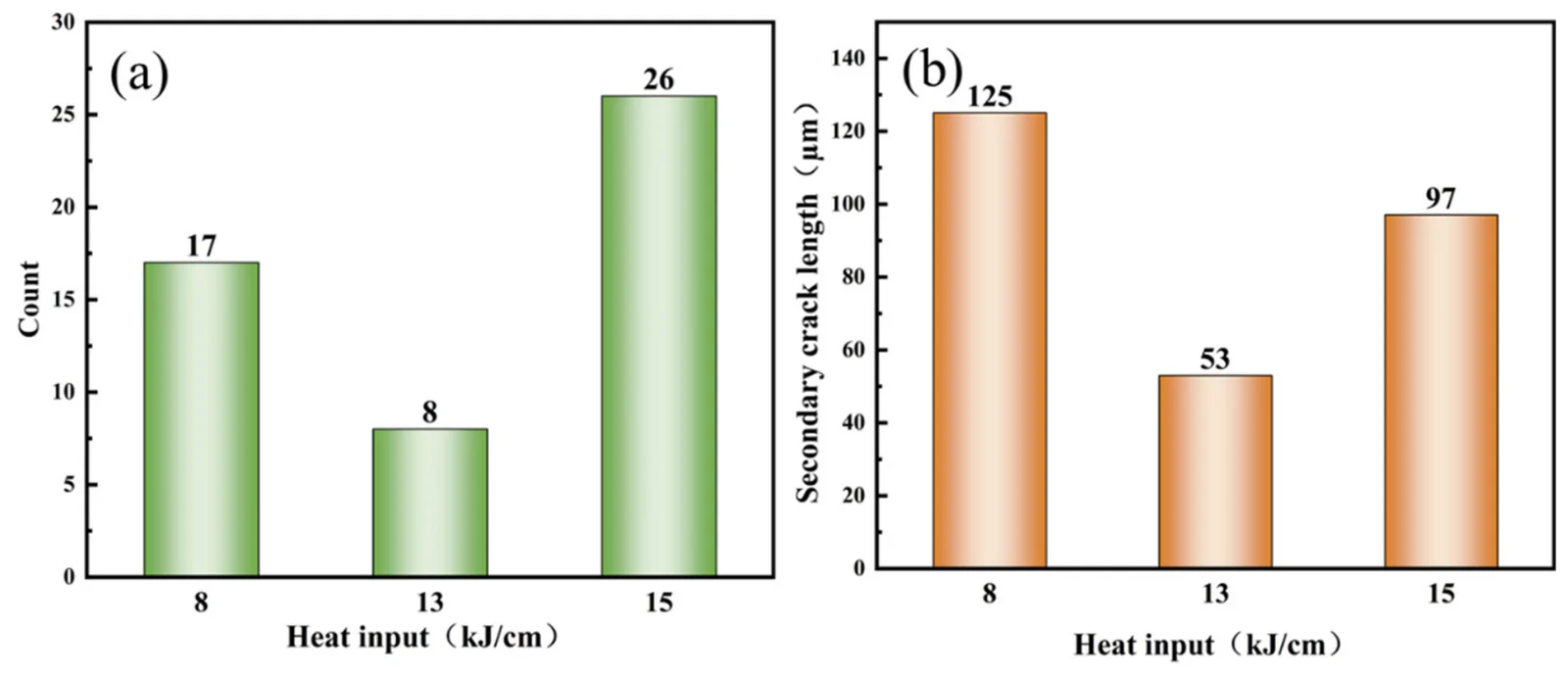
Quantitative evaluation of secondary cracks in 0.5Cr experimental steel: (**a**) number of secondary cracks, (**b**) average length of secondary cracks.

The number and average length of secondary cracks within the identical observation area of the 0.5Cr experimental steel were statistically quantified under heat inputs of 8 kJ/cm, 13 kJ/cm and 15 kJ/cm, and the statistical results are presented in Figure 19. It can be observed that the specimen subjected to 8 kJ/cm heat input possesses fewer cracks but a larger average crack length compared with the sample treated at 15 kJ/cm heat input. For the steel with 8 kJ/cm heat input, the microstructure consists of bainitic ferrite (BF) with a low fraction of M/A constituents, which restrains crack initiation effectively. Nevertheless, cracks can propagate straightly along the direction parallel to BF laths, lengthening the overall crack propagation path. In contrast, the microstructure transforms into granular bainite (GB) at 15 kJ/cm heat input. Numerous hard and brittle M/A constituents inside granular bainite readily trigger microcrack nucleation; however, cracks will be arrested once their propagation direction is perpendicular to part of the strip-shaped M/A constituents, consequently shortening the propagation path. Combined with the fact that the low-temperature toughness at 8 kJ/cm heat input is remarkably superior to that at 15 kJ/cm, it is indicated that M/A constituents exert a dominant detrimental effect on toughness degradation in the coarse-grained heat-affected zone (CGHAZ). This conclusion agrees well with the findings reported in the literature [37,38]. In comparison, both the crack number and average crack length reach the minimum value at the heat input of 13 kJ/cm, demonstrating that grain refinement effectively suppresses both the initiation and propagation of secondary cracks simultaneously.

### 4.2. Analysis on the Effect of Welding Heat Input on M/A Constituents in CGHAZ

During phase transformation upon cooling after pipeline steel welding, when ferrite transformation occurs in supercooled austenite, the solubility limit of carbon in ferrite drives carbon atoms to diffuse into the surrounding austenite zones, forming locally carbon-enriched regions. As the temperature keeps decreasing and bainitic laths start nucleating and growing, part of these carbon-rich austenite regions cools down to the critical temperature for martensite transformation and transforms into martensite. The remaining high-carbon austenite fails to complete full transformation due to insufficient kinetic conditions and is retained at room temperature in a metastable state, eventually forming island-shaped M/A constituents composed of martensite plus retained austenite. Differences in this phase transformation sequence result in the characteristic distribution of multiphase microstructures.

The effects of different types of M/A constituents on crack propagation mainly depend on their morphology, spatial distribution, hardness and interfacial bonding strength [39,40,41]. Elongated M/A constituents generally distribute as continuous or ductile bands. Their weak interfacial bonding enables cracks to propagate directly through these zones without obvious deflection or detour. The continuity and low interface strength of elongated M/A reduce the energy required for crack growth, facilitating fast crack extension along these regions. In extreme cases, elongated M/A constituents can serve as rapid crack propagation channels and induce sharp toughness deterioration. Spherical dot-like M/A constituents exist as isolated and dispersed particles. Owing to their high hardness and strength, propagating cracks deflect slightly and bypass these hard particles. Such particles twist the crack path, raising energy consumption and improving fracture toughness. Nevertheless, superposition of stress fields around adjacent dot-like M/A constituents may trigger local stress concentration and initiate microcracks or secondary cracks. Blocky M/A constituents present as large lumps or clusters. Their large size and high hardness force incoming cracks to undergo prominent deflection or detour, substantially elevating energy dissipation during crack growth and enhancing toughness. However, local fracture or interfacial debonding tends to take place at the boundaries between blocky M/A and the matrix, generating micro-voids. Finely dispersed M/A constituents are uniformly distributed tiny particles in the matrix. They hinder crack propagation homogeneously and improve overall material toughness by blunting crack tips and alleviating stress concentration to retard crack growth. Cracks have to bypass numerous fine M/A particles during propagation, consuming extra deformation energy. In addition, chain-like M/A constituents continuously line along prior austenite grain boundaries or bainite lath boundaries, and film-like M/A constituents form thin layers sandwiched between bainitic laths with thickness generally less than 0.5 μm [42].

**Figure 20:**
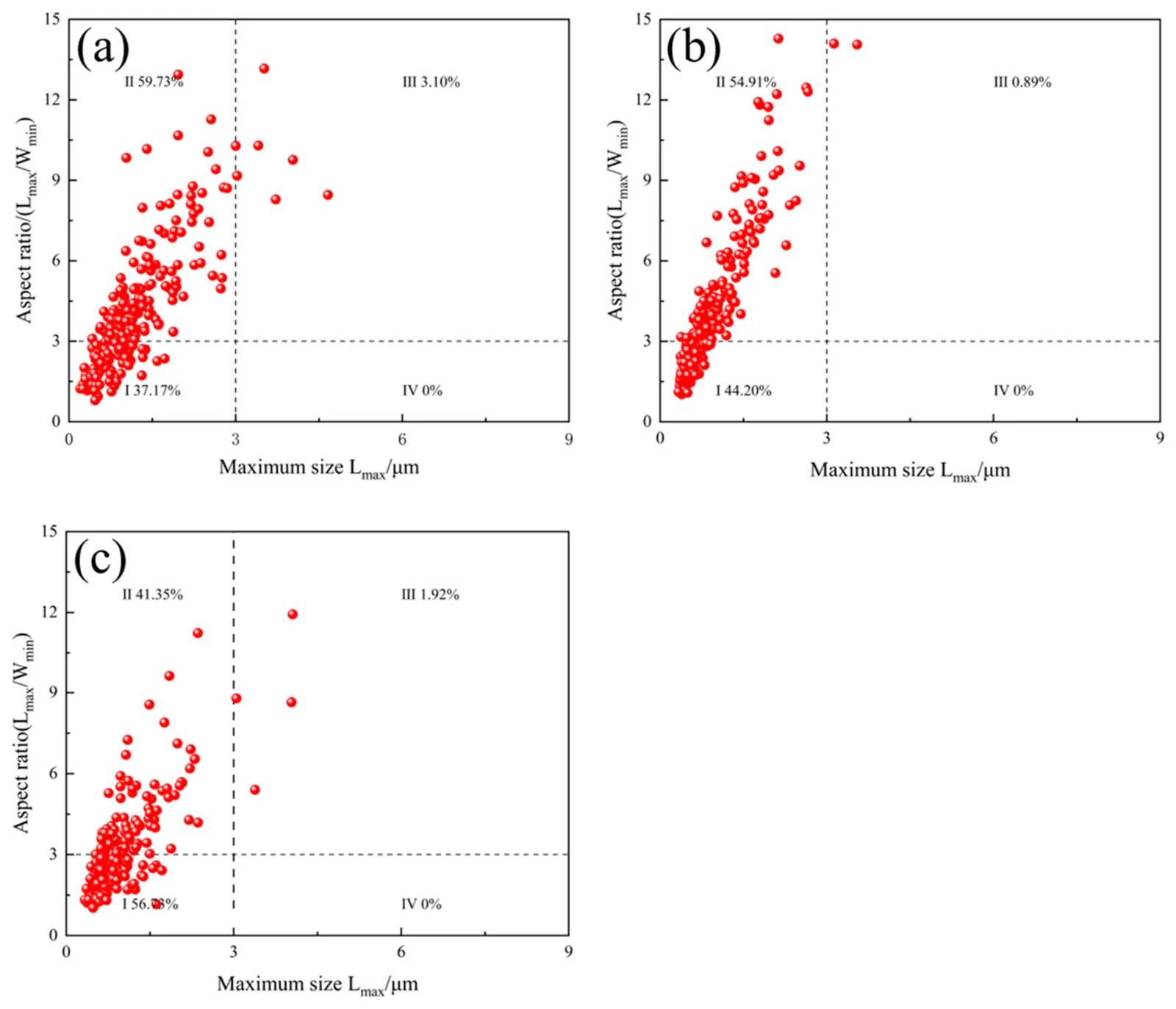
M/A component distribution of different welding heat inputs: (**a**) 8 kJ/cm, (**b**) 13 kJ/cm, (**c**) 15 kJ/cm.

The 3 μm threshold length adopted herein is a widely accepted criterion for quantitatively distinguishing M/A constituents in low-carbon low-alloy weld CGHAZ, which has been validated in multiple previously published studies focusing on martensite-austenite island characterization [43]. Image-J software (https://imagej.net/ij/ (accessed on 4 August 2026)) was adopted to measure and statistically analyze the SEM micrographs of the 0.5Cr steel obtained at welding heat inputs of 8 kJ/cm, 13 kJ/cm and 15 kJ/cm. For each experimental condition, ten randomly selected and non-overlapping microscopic fields were analyzed to guarantee statistical representativeness. All micrographs used for ImageJ quantitative analysis were captured at a consistent magnification of 5000×, ensuring uniform resolution and comparable statistical accuracy among different groups. Using a threshold length of 3 μm and the aspect ratio of individual M/A constituents as classification criteria, M/A constituents were categorized into four types, Type I (dot-like), Type II (elongated), Type III (coarsely elongated) and Type IV (blocky), according to the zone-proportion statistics from Figure 20, the welding heat input significantly alters the total content of harmful M/A constituents (Zone II + Zone III). At a heat input of 8 kJ/cm, the total proportion of slender and coarse elongated M/A reaches 62.83%, among which coarse M/A particles larger than 3 μm occupy 3.10%. When the heat input increases to 13 kJ/cm, the total fraction of harmful M/A decreases to 55.80%, and the proportion of coarse M/A is merely 0.89%. Further raising the heat input to 15 kJ/cm reduces the total fraction of harmful elongated M/A to 43.27%, with coarse M/A accounting for 1.92%.

With increasing heat input, the volume fraction of elongated M/A constituents declines while that of dot-like M/A rises in the 0.5Cr steel. At 13 kJ/cm, elongated M/A still dominates alongside an increased fraction of dot-like M/A; coarsely elongated M/A nearly disappears, and interwoven M/A structures improve the fracture resistance of the material (Figure 5c and Figure 8c). When the heat input further rises to 15 kJ/cm, the growing proportion of dot-like M/A fails to promote toughness improvement. This phenomenon can be attributed to the microstructure characteristic: the granular bainite-dominated matrix possesses a low density of high-angle grain boundaries, which cannot effectively block crack propagation.

## 5. Conclusions

(1) For deep-sea oil and gas transmission pipeline steels, the applicable welding heat input range of experimental steels gradually decreases with the increase in Cr content. Specifically, 0.2Cr steel exhibits excellent toughness in the range of 8–20 kJ/cm of heat input. The high-toughness heat input window for 0.5Cr steel is 8–13 kJ/cm, while 0.8Cr steel only achieves superior toughness at a heat input of 15 kJ/cm.
(2) The coupled effects of welding heat input and Cr content on CGHAZ properties are determined by the combined action of microstructure type, phase fraction, substructure and grain size. With increasing heat input and Cr content, the fraction of lath-like bainites with desirable toughness decreases, while the fraction of large-sized granular bainites with poor toughness increases. Meanwhile, the effective grain size of the whole microstructure presents a trend of first decreasing and then increasing.
(3) At a fixed heat input, the increase in Cr content improves steel hardenability. The well-developed lath bundle structure of BF is beneficial for toughness improvement, whereas the elevated phase transformation stress plays an adverse role for low-temperature toughness. In addition, the amount of the M/A component in the microstructure increases significantly, eventually worsening the overall toughness. For steel with the same Cr content, the prior austenite grain size is enlarged, the BF lath bundle is broadened, a partial GB is formed, and the fraction of M/A constituents in the GB increases as the weld heat input rises. Nevertheless, the above microstructural evolution shows a clear difference in the degree of variability with increasing Cr content.
(4) For the 0.2Cr experimental steel, the broadening degree of BF lath bundles is limited, and M/A constituents in GB are fine, thereby maintaining a high toughness level throughout the whole tested heat input range. For the 0.5Cr experimental steel, BF possesses a more highly developed lath structure, accompanied by a simultaneous increase in the phase transformation stress. Its overall toughness is lower than that of 0.2Cr steel under the coupled effect of both factors. At the heat input of 13 kJ/cm, the microstructure consists of BF and fine-grained GB. The ferrite matrix grains of fine GB are refined with irregular grain boundaries, which significantly refines the effective grain size of the whole microstructure. When the heat input is increased to 15–20 kJ/cm, the formation of GB dramatically increases the M/A fraction, resulting in a sharp drop in toughness. For the 0.8Cr experimental steel, the hardenability is further enhanced, and the number of M/A constituents in microstructure is significantly increased, leading to a severe deterioration of the overall toughness.
(5) Considering the operational requirements of deep-sea pipeline engineering and current limitations in research, further in-depth studies could be conducted in areas such as multi-factor coupled service performance, alloy system optimization, welding process improvement and expansion.

## Figures and Tables

**Table 1:** Chemical composition of pipeline steel (wt. %).

Steel	C	Si	Mn	Cr	Ni + Mo + Cu	Nb	Ti
0.2Cr	0.05	0.20	1.31	0.21	≤0.70%	0.039	0.011
0.5Cr	0.05	0.20	1.32	0.50	≤0.70%	0.038	0.012
0.8Cr	0.05	0.20	1.31	0.79	≤0.70%	0.039	0.011

**Table 2:** CGHAZ under different welding heat input thermal simulation parameters.

Heat Input (E/kJ/cm)	Heating Rate (°C·S^−1^)	T_8/3_ (s)	Peak Temperature (°C)
8	130	10.30	1350
10		12.88	
13		16.74	
15		19.31	
20		25.75	

**Table 3:** Mechanical properties of base metal pipeline steel.

Steel	Rt_0.5_/MPa	R_m_/MPa	Rt_0.5_/Rm	A/%	HV0.5	−10 °C Impact Energy/J
0.2Cr	501	615	0.81	25.0	215	277 ± 7.3
0.5Cr	528	658	0.80	23.0	221	235 ± 5.2
0.8Cr	455	683	0.67	24.5	232	205 ± 5.6

## Data Availability

The original contributions presented in this study are included in the article. Further inquiries can be directed to the corresponding author.
